# Emerging Trends in Nanotechnology and AI-Driven Valorization of Agro-Industrial Waste in Circular Bioeconomy for Production of Biostimulants

**DOI:** 10.3390/foods15132274

**Published:** 2026-06-25

**Authors:** Ikhlas Laasri, Vaibhav Shrivastava

**Affiliations:** 1 BETA Tech Center (TECNIO Network), University of Vic-Central University of Catalonia, Ctra. De Roda 70, 08500 Vic, Spain; 2 Department of Energy and Technology, Swedish University of Agricultural Sciences, Lennart Hjelms väg 9, Box 7032, 750 07 Uppsala, Sweden

**Keywords:** circular bioeconomy, sustainable intensification, precision agriculture, agricultural policy, soil carbon sequestration, biomass upcycling, smart delivery systems

## Abstract

The global agricultural sector faces the dual challenge of increasing productivity while mitigating environmental impacts caused by synthetic agrochemicals and massive agro-industrial waste. This review examines the transition to “Biostimulants 4.0,” a circular economy paradigm driven by the valorization of biomass residues into high-value biological inputs through nanotechnology and Artificial Intelligence (AI). Our analysis highlights that green extraction methods, specifically enzymatic hydrolysis, preserve bioactive integrity and reduce carbon emissions by up to 23.2 times compared to synthetic nitrogen production. Furthermore, waste-derived formulations and nanoscale smart-delivery systems dramatically enhance crop performance; for instance, chitosan nanoparticles can achieve up to a 471% increase in specific growth metrics through sustained-release pathways. To move the industry beyond empirical trial-and-error, the integration of AI-driven predictive models now achieves up to 87% accuracy in forecasting biostimulant efficacy. Finally, we contrast global regulatory frameworks and evaluate the monetization of biostimulant-driven carbon sequestration, capable of generating high-integrity credits priced up to $35 per tonne, as a critical economic pathway to accelerate commercial adoption and incentivize a resilient, decarbonized agricultural system.

## 1. Introduction

The increasing global demand for food, together with the environmental impacts of intensive agricultural practices, has intensified the search for more sustainable production systems. To meet the Zero Hunger target SDG2 of the 2030 UN Agenda for Sustainable Development, agricultural productivity must double by 2030 [1]. Conventional reliance on mineral fertilizers and agrochemicals has contributed to soil degradation, nutrient losses, and greenhouse gas emissions; currently, up to 40% of the world’s land is degraded, a crisis that directly reduces crop yields for 1.7 billion people [2]. Projections from the UN warn that if intensive chemical-based practices persist, 95% of Earth’s soils could be degraded by 2050, fundamentally threatening long-term agroecosystem resilience [3].

Addressing these challenges requires not only improving input efficiency in crop production but also rethinking how resources are generated, used, and recovered across the entire food system. The global food system is characterized by substantial systemic inefficiencies, most notably the generation of approximately 1.3 billion tonnes of food loss and waste (FLW) annually, representing roughly one-third of all food produced for human consumption [4]. This loss extends beyond caloric waste to a critical squandering of embedded resources: FLW accounts for 8–10% of global greenhouse gas emissions, occupies a land area larger than China (28–30% of total agricultural land), and consumes nearly one-quarter of all agricultural freshwater [2]. Currently, much of this biomass ends up in landfills, contributing to 20% of human-caused methane emissions, a gas with 80 times the warming potential of CO_2_ over a 20-year period [5]. The global food waste crisis, which accounts for over 1 billion tonnes of waste annually, represents an urgent sustainability imperative with profound environmental and economic repercussions. In the European Union, over 58 million tonnes of food waste (equating to 130 kg per inhabitant) are generated annually, with an estimated market value of €132 billion [6]. Households remain the primary contributors, responsible for 53% of this total, while the remaining 47% originates across the supply chain, spanning manufacturing (19%), food services (11%), primary production (10%), and retail (8%) [6]. Similarly, the United States generates approximately 60 million tons of food waste each year, representing 30–40% of its total food supply; as the largest component of municipal solid waste in landfills, this decomposing matter acts as a potent source of methane emissions [7,8].

The challenge is equally critical in emerging economies. China reports an annual FLW rate of 22.7%, amounting to approximately 460 million tons [9]. Meanwhile, the Middle East and North Africa (MENA) region generates over 155 million tons of waste annually, a figure projected to nearly double to 294 million tons by 2050 [10]. In this region, where less than 10% of waste is recycled or composted, mismanagement results in US$7.2 billion annual environmental damage, further exacerbating soil and water pollution [10]. These figures underscore a systemic failure in resource efficiency, necessitating a rapid transition toward circular bio-economies that can transform these non-valorized byproducts into high-value agricultural inputs.

Rather than an environmental burden, these streams constitute abundant, underutilized biomass reservoirs, including 449 million tons (MT) of fruits and vegetables, 25 MT of aquatic FLW, and 261 MT of roots, tubers and oil crops generated annually that can be redirected toward the production of value-added agricultural inputs [11]. By valorizing these nutrient-rich feedstocks, such as plant residues and animal processing byproducts, the agroscience market is driving the implementation of eco-friendly practices that support long-term sustainability through plant biostimulants [12]. Additionally, the escalating frequency of extreme climate events, exacerbated by intense El Niño-driven weather patterns, poses an unprecedented threat to global food security [13]. To safeguard productivity against such volatility, the transition to biostimulant-driven, climate-resilient agriculture has become an urgent imperative [14]. This paradigm shift is intrinsically linked to the United Nations Sustainable Development Goals; specifically, valorizing agro-industrial waste into advanced biological inputs directly advances SDG 12 (Responsible Consumption and Production) [15]. Furthermore, by providing a physiological buffer to maintain yields under severe environmental stress and substituting emission-heavy synthetic fertilizers, these bio-based inputs simultaneously drive both SDG 2 (Zero Hunger) and SDG 13 (Climate Action) [15]. While these products have become leading solutions for sustainable intensification, their inherent complexity means that efficacy studies remain challenging, as their beneficial effects are often the result of synergistic interactions rather than a single component [12].

Specifically, a plant biostimulant is defined as any substance or microorganism applied to plants or the rhizosphere that stimulates natural processes to enhance nutrient use efficiency (NUE), abiotic stress tolerance, and crop quality, regardless of its nutrient content [8]. The valorization of such food and agro-industrial waste through targeted extraction and conversion processes aligns with circular economy principles, transforming underutilized organic matter into functional agricultural inputs. This approach not only reduces the environmental footprint of agricultural systems but also generates economic value from novel bio-based products while contributing to improved food quality and enhanced human and environmental health [16].

The economic value of the biostimulant market is substantial and growing rapidly. Recent industry analyzes value the global biostimulants market size has reached to $4.23 billion in 2025, with projections suggesting it will grow to $6.43 billion in 2030, driven by an expected compound annual growth rate of 8.8% [17]. These figures reflect increasing farmer adoption driven by sustainability goals, organic production systems, and climate change pressures. The broader food waste valorization market, valued at $77.6 billion in 2024 and expected to grow to $132 billion by 2034, serves as a powerful economic catalyst. This rapid growth, maintaining a steady CAGR of 5.5% to 6.6% [18], highlights a clear financial incentive for redirecting underutilized biomass into the production of high-value agricultural biostimulants.

Regionally, Europe holds the largest market share at approximately 45%, while North America and Asia represent about 20% each, and Latin America accounts for roughly 15%. The European market has shown significant historical growth, rising from a value of USD 200–400 million in 2011 to USD 500 million in 2013, and is forecasted to reach USD 1500.79 million by 2025. France, Italy, and Spain are the primary European producers. In North America, the market was valued at USD 0.27 billion in 2011. Data from 2013 valued the Latin American market at USD 0.16 billion and the Asia-Pacific market at USD 0.25 billion. More recently, the Australian biostimulants market was estimated at USD 233.8 million in 2015 [19]. On a global scale, most protein hydrolysates (PHs) for agricultural use are produced by companies located in Italy, Spain, the United States, China, and India [20].

While recent literature has extensively mapped circular bioeconomy pathways, such as converting industrial waste into biofuels or AI-driven green catalysts [21], the valorization of these feedstocks into agricultural biostimulants represents a uniquely closed-loop solution that directly addresses global food security. To address current gaps in the literature, this review adopts an end-to-end systems approach that encompasses the entire biostimulant value chain, from the biotechnological upcycling of food waste to precision production and end-user adoption. The primary novelty of this work lies in its cross-disciplinary synthesis: it bridges technical production pipelines (contrasting conventional versus “green” extraction methods) with a detailed examination of global regulatory landscapes frequently overlooked in standard reviews (Appendix A). Furthermore, this paper uniquely integrates the socio-economic realities of farmers, addressing adoption barriers like “initial input shock” and the need for “yield insurance”, alongside a novel framework for decarbonization finance [21]. By demonstrating how biostimulant-driven nutrient use efficiency can be commercialized through soil carbon credits, this review outlines how discarded biomass can be transformed into verified climate assets, providing a previously unconsolidated strategic roadmap for researchers and policymakers.

## 2. Concept and Definition of Biostimulants

According to the EU Regulation (2019/1009), biostimulants are classified into two main groups: microbial and non-microbial biostimulants.

### 2.1. Microbial Biostimulants

Microbial biostimulants comprise beneficial microorganisms that colonize the rhizosphere or plant tissues to promote growth through various biological mechanisms, including biological nitrogen fixation, the solubilization of phosphorus and potassium, the production of phytohormones like auxins, and the induction of systemic resistance against pathogens (Table 1). These products are typically standardized based on viable cell metrics such as colony-forming units, spore counts, or filamentous biomass and require carefully controlled formulation and storage to preserve viability [22].

Under the EU Fertilizing Products Regulation (FPR), microbial biostimulants are classified as Product Function Category (PFC) 6(A). Although the Plant-Growth-Promoting Rhizobacteria (PGPR) category includes a wide array of genera such as *Bacillus* and *Pseudomonas*, Regulation (EU) 2019/1009 currently restricts authorized biostimulants to *Azospirillum*, *Azotobacter*, and *Rhizobium*, alongside arbuscular mycorrhizal fungi (AMF) like *Glomus* and *Rhizophagus* spp. [23]. The regulation permits these products to contain dead cells or non-harmful production residues, provided they belong to these specific taxa and have undergone minimal processing, such as drying or freeze-drying.

While these microorganisms were originally selected for their phenotypic traits, notably nitrogen fixation and phosphorus solubilization, the number of studies investigating microbial consortia is growing exponentially. Advances in high-throughput sequencing and microbial taxonomy have revealed limitations in traditional classifications, underscoring the urgent need to reassess and expand the list of eligible microorganisms under the current EU framework [24].

**Table 1:** Summary of recent research demonstrating the efficacy of specific microbial biostimulants on the yield and quality parameters of selected high-value crops.

Plant	Species	Effect	Reference
Cannabis	*Mucilaginibacter* sp.	+24% Flower dry weight (yield), 11.1% Total CBD, 11.6% Total THC	[25]
	*Pseudomonas* sp.	+28% Stem dry matter, 7.2% Total CBD, 5.9% Total THC	
*Rubus idaeus* L.	*Bacillus subtilis* and/or *Paenibacillus* sp.	Increase in antioxidant content (16–20% and in degree of Methylation by 9% to 44%)	[26]
*Fragaria ananassa* Duch.	*Bacillus subtilis* and *Paenibacillus polymyxa*	Increase in Soluble Solid Content by 14% and in Anthocyanin Content by 27%	[27,28]
Strawberry	*Claroideoglomus etunicatum*	Increased sugar content by 28% and the sugar/acid ratio by 31%.	[29]
	Multispecies mycorrhizal community	Increase in anthocyanin content by 39%	
	*T. harzianum + Claroideoglomus etunicatum*	Increase in flavonoid content 41%	
*Helianthus tuberosus* L.	*Klebsiella variicola*, *Rhizophagus intraradices*	Increase in tuber inulin content by 66.7% and 150% in plant height	[30]

Bacillus-based biostimulants are among the most widely used microbial products due to their efficient production of bioactive metabolites and their ability to form endospores, which confer high stability and viability in commercial formulations [31]. Nevertheless, current research efforts are expanding beyond *Bacillus* spp. to identify novel microorganisms with biostimulant potential that can meet agronomic performance and market demands. Microbial biostimulants promote plant growth and development by producing phytohormones involved in growth regulation, flowering, and yield formation. In addition, they synthesize vitamins that enhance plant resistance to pathogenic bacteria, viruses, and fungi, while facilitating atmospheric nitrogen fixation and nutrient mobilization [32].

### 2.2. Non-Microbial Biostimulants

Non-microbial biostimulants are defined as distinct organic or inorganic substances, or mixtures thereof, that stimulate natural plant processes independently of the product’s inherent nutrient content [22]. The primary function of these substances is to enhance nutrient uptake, improve nutrient use efficiency (NUE), increase tolerance to abiotic stress, and elevate overall crop quality traits [14]. According to standard regulatory and scientific frameworks (e.g., Regulation (EU) 2019/1009), they operate not as traditional fertilizers, but by modulating plant physiological and molecular pathways.

#### 2.2.1. Seaweed Extract

Seaweeds have emerged as primary natural biostimulants, categorized by pigmentation into Phaeophyta (brown), Rhodophyta (red), and Chlorophyta (green). These extracts contain a complex assembly of macro- and micronutrients, trace elements, polysaccharides, vitamins, and hormone-like compounds that synergistically promote root development, improve nutrient uptake, and enhance tolerance to both abiotic and biotic stresses [33]. Notably, extracts from Phaeophyta, such as *Sargassum* spp., have shown particularly strong biostimulatory effects. The massive accumulation of Sargassum biomass along coastlines now presents a strategic opportunity to valorize this abundant resource into high-value agricultural products, transforming a significant environmental burden into a sustainable economic asset [34].

This shift towards seaweed-based biostimulants offers a viable alternative to synthetic agrochemicals, facilitating integrated crop management strategies that reduce pesticide and fertilizer inputs while simultaneously boosting productivity. Such innovations align with circular economy principles and climate-resilient agriculture frameworks. Driven by these advancements, Europe currently leads the global market, commanding a 42.6% share of total consumption as of 2024–2025 [35]. This regional dominance is largely attributed to stringent environmental regulations, such as the EU Fertilizing Products Regulation (2019/1009), and the widespread adoption of sustainable and organic farming practices across the continent.

#### 2.2.2. Protein Hydrolysates

PHs represent a promising category of biostimulants, with their composition ranging from free amino acids to oligo- and polypeptides varying according to the raw material and the hydrolysis method employed, which are manufactured from protein sources using partial hydrolysis [12]. Amino acids play a dual role in plants, serving both as fundamental building blocks of proteins and as readily available sources of organic nitrogen. This dual function contributes to the mitigation of abiotic stresses such as drought and salinity, while also promoting cell growth and metabolic activity. Due to their structural role as protein units, amino acids are essential for plant metabolism, growth, and development [36]. PH-based biostimulants are usually extracted from protein-rich agro-wastes, either from animal byproducts (e.g., leather by-products, feathers, blood meal, fish, and casein) and plant biomass (e.g., legume seeds, alfalfa hay, corn wet-milling residues, and vegetable by-products) [20].

A major trend in recent years is the valorization of protein-rich agro-industrial waste, transforming disposal problems into sustainable inputs that drive plant growth under normal conditions. For instance, application of 7.5% hempseed-derived protein hydrolysate significantly boosted Red Oak lettuce yield, achieving a shoot fresh weight of 148.00 g/plant and increasing chlorogenic acid levels by approximately 3.77 times compared to the control. Similarly, treating olive seedlings with pumpkin seed-derived H2 hydrolysate enhanced shoot height by 20–30% and optimized physiological performance through a superior amino acid profile rich in L-proline and L-arginine [37]. Similarly, in the realm of animal byproducts, a study focused on feather waste valorization, biodegradation by *Gordonia alkanivorans* S7 achieved a 99% mass loss of household chicken feathers after 168 h of cultivation. The resulting hydrolysate, containing 8.9% nitrogen and 31.2% carbon, significantly stimulated plant growth, reaching maximum root growth stimulation of 31% for *Lepidium sativum* and 26% for *Sorghum saccharatum* [38]. Furthermore, aquatic waste streams have proven equally effective; Nuzhyna et al. [39] extracted PH from rainbow trout processing waste (specifically fish guts) and applied them to agricultural crops. The results showed that FPH treatments led to a 15–22% increase in plant biomass and enhanced NUE, specifically boosting nitrogen accumulation in leaves by up to 18% compared to untreated plants.

#### 2.2.3. Humic Acids

Humic substances, known as the black gold of agriculture, originate from the microbial decomposition and chemical transformation of dead biota in soils. They are regarded as the most abundant naturally occurring organic compounds on earth and constitute a major fraction of soil organic matter (80%). Humic products derived from renewable sources such as byproducts of agro-industrial processing or animal husbandry exhibit high bioactivity and represent a sustainable alternative to conventional humic products obtained from coal, lignite, or peat [40]. In soils, humic substances play crucial roles in regulating nutrient availability, facilitating carbon and oxygen exchange between the soil and the atmosphere, and influencing the transformation and mobility of toxic compounds. Moreover, they directly affect plant physiological processes and shape the composition and functionality of rhizosphere microbial communities [41]. They are commonly classified according to their solubility characteristics as follows: (i) humins, which are insoluble in water and cannot be extracted from soil; (ii) humic acids (HA), which are soluble in water under alkaline conditions; and (iii) fulvic acids, which remain soluble in water across the entire pH range. HA can be extracted by alkaline extraction, as it was mentioned in the study by Nardi et al. [42]. The application of HA fraction (HEf) at 1 mg C L^−1^ induced a strong auxin-like response in *Nicotiana plumbaginifolia* leaf explants, promoting root formation, whereas untreated controls developed no roots. HEf increased protein content by ~51% and peroxidase activity by ~67% relative to the control, and produced longer roots with fewer root hairs than those induced by indole-3-acetic acid (IAA), demonstrating a clear stimulatory effect on plant growth and differentiation. In another study by Qin et al. [43], the application of HA by concentration of 200 mg L^−1^. At this dose, root biomass increased by ~35–40%, shoot growth by ~25–30%, and nutrient uptake by up to ~25%, alongside a ~30% increase in chlorophyll content. These findings are consistent with commercial applications, such as Grow-plex SP, a HA–based biostimulant that promotes soil microbial activity, enhances root and shoot development, and improves iron and zinc uptake in plants.

#### 2.2.4. Chitin and Its Derivatives

Chitin and its deacetylated derivative, chitosan, are increasingly valued as sustainable biostimulants extracted from the vast agro-industrial waste streams of the seafood industry (crustacean shells). Beyond their traditional role in defense priming, chitosan-based biostimulants have been recently quantified for their ability to significantly enhance crop yields by improving soil health, optimizing water and nutrient availability, and modulating the soil microbiome [44]. For instance, a study by Assali et al. [45] synthesized dual-functionalized tryptophan-lysine and valine-lysine chitosan biostimulants that achieved a 471% increase in stem length and a 300% expansion in root length in *Hordeum vulgare*. These specific amino acid grafts allow the matrix to act as a sustained-release reservoir, enhancing drought resilience by boosting photosynthetic efficiency by 15.6% and maintaining 55.37% higher moisture content. Consequently, these functionalized polymers serve as sophisticated delivery systems that optimize both physiological regulation and water use efficiency for sustainable agriculture. Another study on salicylic acid-functionalized chitosan nanoparticles significantly enhanced maize productivity, achieving a 57.8% increase in grain yield. This nano-formulation further serves as a potent biostimulant for disease control, reducing the incidence of post-flowering stalk rot by 59.4% through mechanisms such as antioxidant enzyme elevation and lignin-based cell wall reinforcement [46].

#### 2.2.5. Inorganic Compounds

Inorganic compounds in agriculture refer to beneficial elements (such as Silicon, Selenium, Cobalt) and inorganic salts that are not classified as essential macronutrients but are crucial for strengthening plant cell walls, enhancing metabolic efficiency, and inducing stress tolerance. In modern applications, they are often engineered as nanoparticles or conjugated with polymers to improve their stability and absorption [47]. A study on comprehensive silicon treatments involving seed dressing and foliar sprays significantly enhanced spring wheat productivity, increasing grain yields by an average of 8.9% and 7.6% [48].

Current literature on non-microbial biostimulants is overly focused on short-term success metrics, such as percentage yield gains, at the expense of scientific rigor. This empirical bias fails to address three systemic bottlenecks: (1) the lack of standardized extraction protocols for heterogeneous feedstocks, rendering results difficult to reproduce; (2) an absence of techno-economic analysis (TEA) to justify the scalability of high-performance engineered inputs; and (3) a reliance on controlled-environment data that ignores the stochastic pressures of the field. To evolve, the sector must prioritize standardized ingredient profiling and multi-environmental validation over isolated efficacy trials [27].

The reported performance of non-microbial biostimulants exhibits significant heterogeneity, reflecting the diverse crop species, environmental stressors, and application methods employed across studies [27]. To contextualize these findings, efficacy must be normalized across three primary variables: Environmental Stochasticity, distinguishing between the stable conditions of controlled greenhouse experiments and the unpredictable stressors of open-field agriculture; application delivery Efficiency, identifying the distinct bioavailability outcomes of foliar versus soil-drench or seed-priming methods; and Phenotypic Baseline Sensitivity, which differentiates between biostimulants aimed at vegetative growth versus those designed for abiotic stress adaptation [27]. Interpreting these results through this framework reveals that reported percentage increases are not universal metrics, but context-dependent outputs. Future research must prioritize multi-environmental trials that account for these variables, moving away from isolated, single-location efficacy reporting to provide the standardized comparative data required for commercial-scale predictive modeling.

### 2.3. Synergistic Effects: Microbial and Non-Microbial Consortia

While the individual categories of microbial and non-microbial biostimulants offer distinct pathways for growth, the frontier of biostimulants moves beyond isolated applications toward integrated synergistic systems. By combining these diverse biological tools, researchers are creating consortia that mimic natural ecosystems to overcome the “greenhouse-to-field” gap and ensure consistent agronomic performance. In these consortia, non-microbial components often act as “prebiotics,” providing the necessary carbon and energy sources for “probiotic” microbes to establish within the rhizosphere.

The “Prebiotic and Probiotic” model in agriculture functions as a synbiotic system, where the non-microbial component provides a competitive advantage to the microbial inoculant. For instance, recent research on tomato demonstrated that the co-application of *Trichoderma harzianum* (probiotic) with *Ascophyllum nodosum* (prebiotic seaweed extract) significantly enhanced fruit yield by 24.8% [49]. Similarly, the application of microcapsules containing *Bacillus megaterium* supplemented with humic acid resulted in the highest reduction in nematode parameters, specifically decreasing the number of galls by 87.2%, egg masses by 88.9%, and second-stage juveniles in soil by 91%. Furthermore, this combined treatment significantly enhanced vegetative growth metrics while simultaneously boosting photosynthetic pigments, antioxidant enzyme activity, and soil microbial enzyme levels [50]. Furthermore, the model has proven effective in mitigating environmental stress. In *Lactuca sativa*, a consortium involving *B. megaterium* and non-microbial biostimulants led to a 17% increase in head weight. The yield boost was driven by a 200% increase in the concentration of the bioactive cytokinin, isopentenyladenosine, which up-regulated genes involved in stress adaptation and protected photosynthetic machinery [51]. These findings suggest that the prebiotic component acts not just as a nutrient, but as a metabolic primer that ensures the “probiotic” microbes can establish and function effectively under field conditions.

## 3. Extraction Methods

Biostimulants can be obtained from a wide range of biological raw materials using different extraction approaches. These methods strongly influence the chemical composition, bioactivity, and agronomic effectiveness of the final product. Broadly, extraction strategies are classified into physicochemical methods and biological methods, depending on whether chemical/physical forces or biological agents are used to release bioactive compounds.

### 3.1. Physicochemical Methods

Physicochemical extraction methods rely on chemical reagents, temperature, pressure, or mechanical forces to solubilize or release bioactive compounds from biomass. These methods are widely used at industrial scale due to their efficiency and reproducibility, although they may require higher energy inputs or chemicals.

#### 3.1.1. Chemical Hydrolysis

Involves treating protein substrates with acids or alkalis to break peptide bonds, yielding free amino acids and short peptides. This method is effective at solubilizing protein but can be harsh and less selective, sometimes degrading sensitive bioactive compounds such as tryptophan, cysteine, serine, and threonine. It is commonly used when rapid and complete hydrolysis is required [20]. Furthermore, this process induces racemization, the conversion of amino acids from the L-form to the D-form, thereby nullifying their biological activity [20].

Chitin was extracted from *Portunus sanguinolentus* using a chemical process involving Hydrochloric Acid (HCl), Sodium Hydroxide (NaOH), and acetone. This extraction method resulted in a chitin recovery yield of 26.15%. Treating seeds and seedlings with a 50% chitin concentration significantly improved germination rates to between 90% for tomato seeds and 95% for Okra seeds, boosted root and shoot lengths by up to 5% and 28%, respectively, and enhanced the plants’ nutritional and medicinal profile through the increased production of beneficial flavonoids, steroids, and glycosides [52].

#### 3.1.2. Organic Solvent Extraction (OSE)

This extraction method, in which plant, algal, or other biological biomass is contacted with organic solvents (e.g., ethanol, methanol, acetone, ethyl acetate, or hydro-alcoholic mixtures) to dissolve and recover low- to medium-molecular-weight bioactive compounds [53]. Meanwhile, 38% of research utilized this technique to extract bioactive compounds from plant biomass. The solvents are subsequently evaporated to ensure the resulting extracts are safe for agricultural application [53]. For example, the Selenium nanoparticles were extracted using a methanolic extract from *Amphipterygium glaucum* leaves. The application of vinca plants increased leaf number by 28% across all doses, while a 50 µM concentration specifically boosted flower fresh weight by 70% and total chlorophyll by 32% [54].

#### 3.1.3. Assisted Extraction Methods

- Supercritical Fluid Extraction (SFE)

It is “green” physicochemical method that uses supercritical carbon dioxide (CO_2_) under high pressure as a solvent. It operates at lower temperatures, preserving volatile and heat-sensitive compounds [55]. Based on the study, the supercritical fluid algal extracts were applied to *Lepidium sativum* L. The most effective treatment was watering after germination with the algal extract, which significantly stimulated root growth to 130% of the control value. For *Triticum aestivum* L., seed maceration extract solution yielded the best results, increasing root weight by 27% and aerial part weight by 19% compared to the control [56]. SFE was used to isolate bioactive compounds from the Baltic macroalgae. The Baltic macroalgae extract was particularly effective on winter wheat, significantly enhancing the mass of 1000 grains by 18.5% compared to the control [57].

- Microwave Assisted Extraction (MAE)

MAE is an innovative, green method for extracting bioactive compounds from algal biomass, offering an efficient alternative to conventional liquid extraction that requires large solvent volumes and multiple steps [58]. For example, biostimulants extracted from *Polysiphonia*, *Ulva*, *Cladophora* and through MAE. The plant height increased by 16% and the chlorophyll content by 12.5% [58].

- Other methods

Additionally, there are other methods used for biostimulants extraction. For example, in research involving *Sargassum horneri*, biostimulants were isolated using boiling, soaking, autoclaving, and ethanol extraction methods to evaluate their effects on the red alga *Neopyropia yezoensis*. The study found that boiling extraction (SBE) was the most effective, significantly increasing algal production by 185% and enhancing superoxide dismutase activity under thermal stress conditions. Additionally, at the optimal temperature of 10 °C, the boiling and soaking methods significantly improved pigment content (phycocyanin and phycoerythrin) compared to other methods [59].

Similarly, extracts were prepared from *Artemisia absinthium* leaves using aqueous methods. The foliar application of cold-soaked extracts and infusions on *Glycine max* (L.) *Merr.* resulted in the highest average yield increases of 31.41% and 23.70%, respectively, compared to the control. Additionally, spraying with infusions increased the number of seeds per m^2^, 43% and the seed fat concentration by 21.40% [60]. These methods effectively recovered bioactive compounds while allowing researchers to differentiate between heat-sensitive and heat-stable fractions. Another green method called Twin-Screw Extrusion through the biostimulants was extracted from sunflower byproducts.

### 3.2. Biological Methods

Biological extraction methods use living organisms or biological catalysts to convert raw biomass into bioactive compounds. These approaches are generally considered more sustainable and selective, preserving functional molecules while aligning with circular economy principles.

#### 3.2.1. Enzymatic Hydrolysis (EH)

EH uses specific proteases (e.g., Alcalase^®^ or microbial proteases), cellulases, or carbohydrases to cleave peptide bonds under controlled conditions of pH and temperature, producing hydrolysates rich in biologically active low-molecular-weight peptides and free amino acids [61]. This approach is often preferred because it can yield compounds with greater bioactivity relevant to plant stimulation and is considered more environmentally friendly due to its low energy requirement and the carbon dioxide emissions. For example, in a study by Shrivastava et al., the biostimulants were extracted from pig slurry through enzymatic hydrolysis and were applied to *Beta vulgaris*. It increased leaf chlorophyll by 10–30%, proline accumulation by up to 60%, and N uptake by ~10–25% [62]. In another study, the extraction of PH from *Agaricus bisporus* was conducted with Alcalase, Flavourzyme and some other enzymes. After applying maize by 1%, the germination rate has increased by 21%, root length by 53% and shoot length by 44% [63]. Additionally, chitosan oligosaccharides were produced via EH and applied to tomato plants (*Lycopersicon esculentum*). This treatment resulted in a 25–30% reduction in disease severity and a mixed treatment has increased by 19–22% the marketable yield during field trials [64].

#### 3.2.2. Fermentation

Fermentation is a process assisted by or involving microorganisms to achieve a value-added product of economic value. This includes the production of microbial biomass, enzymes, and cell metabolites, which serve as either the active ingredients or the biological tools for the final formulation [65]. Submerged fermentation (SmF) is the process in which microorganisms are grown in liquid media to produce microbial biomass, enzymes, organic acids, phytohormones, and metabolites, commonly used for microbial biostimulants. SmF is currently the most widely applied technology. However, depending on microbial requirements, growth media composition, and resource and energy inputs, including large volumes of water, intensive agitation and aeration, and specialized equipment. SmF can be costly and economically unsustainable. In addition, SmF is highly sensitive to operational parameters, prone to contamination, and offers limited control over certain physical and chemical variables, while the recovery of specific enzymes and metabolites can be challenging [65].

For example, Auxym is plant-based commercial biostimulant consisting of tropical extracts generated through water fermentation. Auxym applications have increased the yield of *Diplotaxis tenuifolia* by 11.4%, the leaf area by 12% and the antioxidant content by 15.1% [66]. Solid-state fermentation (SSF), in which bacteria and fungi grow on moist, solid, non-soluble organic substrates under conditions of little or no free-flowing water, has gained increasing attention. Beyond its lower energy requirements and operational advantages over SmF, SSF enables the effective bioconversion of agricultural and industrial organic wastes, thereby supporting circular economy principles and sustainable bioprocessing. In a study, the non-microbial biostimulant was extracted from municipal green waste (grass clippings and wood chips through SSF inoculated with *Trichoderma harzianum*. The resulting biostimulants (Indole-3-acetic acid) increase biomass by up to 189%, chlorophyll a content by 210%, and carotenoids by 175% [67].

The choice of extraction methodology involves a trade-off between technical efficiency, product bioactivity, and sustainability. While physicochemical methods, such as chemical and organic solvent extraction, offer high scalability and rapid results, they often require harsh processing conditions that may compromise the integrity of sensitive bioactive compounds. In contrast, biological methods like enzymatic hydrolysis and fermentation align more closely with circular economy principles by utilizing greener, more selective processes that preserve molecular functionality, albeit often at the cost of longer production cycles or higher operational sensitivity. Ultimately, selecting the optimal approach requires balancing the target biostimulant’s biochemical characteristics against the economic and environmental objectives of the specific production facility.

## 4. Nanobiostimulants and Smart Delivery Systems

The integration of nanotechnology into agriculture has birthed a biostimulants era, characterized by the development of nanobiostimulants, which are not a distinct biological class but rather a formulation technology. This reduction in size significantly increases the surface-area-to-volume ratio, enhancing solubility, stability, and cellular uptake of bioactive formulations engineered at the nanoscale (1–100 nm). By manipulating matter at the atomic level, these formulations enhance the bioavailability of active ingredients, protect them from premature degradation, and facilitate targeted delivery to specific plant tissues [68].

**Figure 1:**
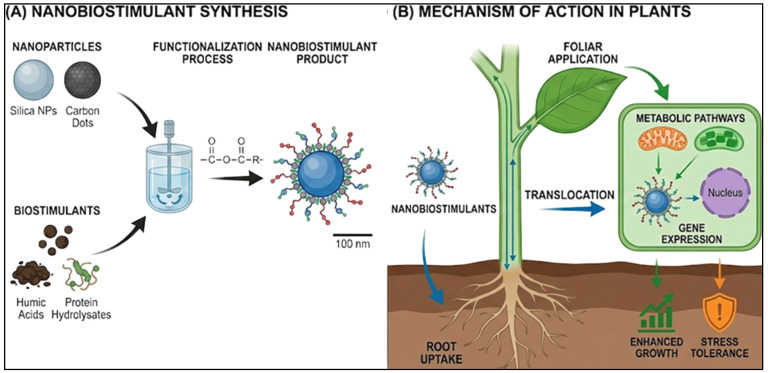
Demonstration of (**A**) The functionalization process of nanoparticles (e.g., Silica NPs or Carbon Dots) with active non-microbial biostimulants (e.g., Humic Acids or Protein Hydrolysates) to create a nanobiostimulant product. (**B**) The biological pathway showing uptake via foliar or root application, followed by translocation to trigger gene expression and metabolic pathways, ultimately resulting in enhanced growth and stress tolerance.

Nanobiostimulants are generally classified into metallic nanoparticles (e.g., Ag, ZnO, TiO_2_), carbon-based nanomaterials (e.g., carbon nanotubes, graphene), and polymeric nanocarriers (e.g., chitosan, dendrimers) [68]. Their efficacy is derived not just from their chemical composition but from their physical ability to penetrate cell walls via apoplastic and symplastic pathways, modulate gene expression, and trigger systemic defense mechanisms at significantly lower dosages than bulk counterparts (Figure 1). Furthermore, the recent shift towards Green Synthesis using plant extracts or microbial enzymes to fabricate these particles has improved the biocompatibility and ecological safety of these inputs.

### 4.1. Smart Delivery Mechanisms

The primary innovation of nanobiostimulants lies in their ability to overcome the physical and environmental barriers that limit conventional inputs. This “smart delivery” capability allows for:

- Nanocarriers can protect sensitive active ingredients (such as amino acids or enzymes) from environmental degradation until they reach the target tissue, facilitating movement through the plant’s vascular system (xylem and phloem) more efficiently than bulk fertilizers.
- Polymer-based nanoparticles can release their payload slowly over time, reducing the washout effect common in traditional soil applications. Modern smart systems are now engineered to trigger release only in response to specific environmental cues, such as rhizosphere pH changes, soil moisture levels, or specific root exudates.
- The enhanced bioavailability means that significantly lower doses are required to achieve the same or superior physiological responses, aligning with sustainable agriculture goals by minimizing chemical runoff [69].

### 4.2. Immunoengineering and Smart Release Mechanisms

One of the most significant advancements in early 2020s is the concept of Plant Immunoengineering using nanoparticles to actively program the plant’s immune system. Conventional foliar sprays often suffer from rapid washout or volatilization. Nanocarriers address this by encapsulating bioactive molecules for controlled, sustained release [70]. For instance, Zhang et al. [70] demonstrated that Salicylic Acid-loaded Silicon Nanoparticles as nano-biostimulants (30–70 nm) to manage twig blight disease in Chinese bayberry. The application of nanobiostimulants reduced disease severity by 47.5%, while simultaneously increasing endogenous salicylic acid by 46.5%, chlorophyll (SPAD) values by 47.3%, and the net photosynthesis rate by 57.6%.

### 4.3. Metabolic Reprogramming and Stress Training

Beyond acting as carriers, the nanoparticles themselves can serve as stress trainers. This mechanism involves exposing plants to a mild, controlled oxidative stimulus (via ROS-generating nanoparticles) that primes the antioxidant defense system without causing damage, effectively “vaccinating” the plant against future stress [70]. Khepar et al. [71] validated this mechanism in rice using Trigolic-formulated Zinc Sulfide Nanoparticles. Nanopriming with these particles significantly bolstered rice seedling vitality by activating antioxidant enzymes, specifically increasing Superoxide Dismutase by 35.47%, Ascorbate Peroxidase by 33.80%, and Catalase by 45.94%.

While the agricultural benefits of nanobiostimulants are significant, their widespread implementation requires a rigorous assessment of the risks associated with their entire life cycle. The production and application phases of nanobiostimulants present risks that are often overlooked, specifically regarding the depletion of natural resources, potential soil and water contamination, and the disruption of local ecosystems through harm to non-target organisms [68]. A critical concern is the persistence and environmental fate of these materials; the potential leaching of nanoparticles into soil and water bodies, coupled with their long-term accumulation, poses significant questions regarding ecosystem health and potential toxicity [68]. Furthermore, the lack of standardized monitoring and regulation complicates our ability to ensure compliance with environmental laws, particularly regarding worker exposure during production and consumer exposure via crop residues. Transitioning to sustainable nano-agriculture requires a holistic approach that moves beyond immediate productivity gains to prioritize standardized safety assessments, proper waste management, and the development of greener fabrication methods that mitigate the long-term impact on biodiversity and soil fertility [68].

While nanobiostimulants and targeted delivery methods offer unprecedented precision, their transition from lab to field is currently hampered by three factors: economic scalability, as high-cost nano-formulations lack the techno-economic analysis required for broad adoption; environmental uncertainty, given the lack of long-term data on nanoparticle soil accumulation; and methodological over-optimization, where success in controlled, ideal conditions ignores the stochastic leaching and adsorption pressures of real-world farming. To reach commercial maturity, the field must prioritize multi-year field validation and LCA frameworks over isolated efficacy trials.

## 5. Application Methods

While the molecular composition of a biostimulant is fundamental, its ultimate efficacy is not solely defined by the type of bioactive compound used; it is also dependent on the method of application. The choice of delivery system, whether foliar spraying, soil drenching, or seed treatment, dictates the entry portal (e.g., leaf cuticle and stomata versus root epidermis) and consequently determines the bioavailability of the active ingredients. Because distinct uptake pathways can trigger different physiological responses, the application strategy must be carefully tailored to the specific environmental constraints and the desired agronomic goals.

### 5.1. Foliar Application (Spraying)

**Figure 2:**
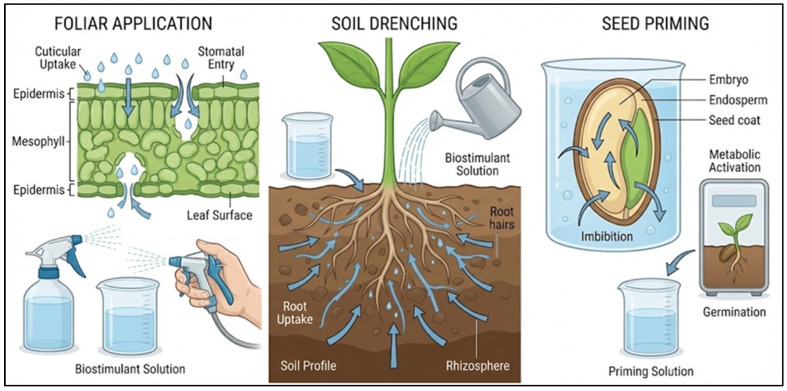
Schematic showing (**left**) Foliar Application through cuticular and stomatal pathways, (**center**) Soil Drenching targeting root uptake within the rhizosphere, and (**right**) Seed Priming to trigger metabolic activation and improved germination.

Foliar application involves spraying the biostimulant directly onto the leaves, allowing for rapid absorption through stomata and the cuticle. Foliar spraying is widely regarded as the most effective method for achieving rapid plant responses, particularly for alleviating temporary stress conditions or nutrient limitations. By applying biostimulants directly to the leaf surface, bioactive molecules can bypass the root system and enter through the stomata and hydrophilic pores (Figure 2) of the cuticle [72]. This method is often preferred for rapid correction of stress or nutrient deficiencies and is frequently more economically efficient [73].

In a comparative study on soybean (*Glycine max*), Szparaga et al. [60] found that Foliar application of *Artemisia absinthium*–based biostimulants significantly increased soybean yield compared with the control, with yield gains of about 31.4% for cold-soaked extracts and 23.7% for infusions. Foliar spraying also resulted in the highest economic benefit, providing an average income of approximately 181 EUR ha^−1^. In another study, the application of Alfalfa PH has increased the total leaf area by 15–30%. The foliar application of chitosan and silicon as biostimulants led to a 20% increase in final berry yield. Additionally, chitosan treatment specifically enhanced fruit quality by increasing pulp firmness by 20%. Furthermore, the use of alfalfa hydrolysate and seaweed extracts significantly improved the fruit’s nutraceutical value, increasing the concentration of phenolic compounds by 18–20% [74].

Additionally, the foliar application of fermented pomegranate waste extract on tomato has boosted crop yield by 34% and enhanced the nutritional quality of the fruit by increasing lycopene content by 32%. Furthermore, the biotechnological process of fermenting pomegranate bagasse with *Aspergillus niger* transformed the substrate itself, increasing its antioxidant activity by 467% and catechin content by 315% [75]. An interval of approximately 10–14 days is recommended to stimulate the best physiological responses [76].

### 5.2. Soil Drenching

Soil applications, including drenching, fertigation, and soil spraying, target the rhizosphere to improve NUE and soil health [77]. Unlike foliar sprays, which provide a transient boost, soil applications often result in sustained benefits by modulating the soil microbiome and improving root system architecture. A comprehensive meta-analysis of 180 field trials suggests that while biostimulants increase yields by an average of 17.9% across all methods, soil-applied treatments often yield the highest potential gains, frequently outperforming foliar and seed applications by an additional 10% in total crop productivity [78]. This is attributed to the ability of soil amendments to buffer physiochemical constraints, such as pH or salinity, before they affect the plant.

For instance, soil drenching has been shown to be superior to foliar sprays in enhancing reproductive traits and improving soil microbial diversity in alkaline environments; in specific cases, drenching with microbial biostimulants has increased seed and oil yields by 124% and 263%, respectively [79]. Furthermore, in controlled environments like hydroponics, the continuous supply of biostimulants (e.g., seaweed extracts or fulvic acids) through the nutrient solution has proven effective in reducing nitrate accumulation in leafy greens like lettuce by 50% while maintaining biomass [80]. Furthermore, supplemental fulvic acid, seaweed extract, and γ-PGA in half-strength Hoagland solution increased lettuce shoot fresh biomass by significant margins, achieving yields comparable to or higher than the 23% increase produced by a full-strength solution.

### 5.3. Seed Treatment (Priming)

Seed priming is an effective and cost-efficient technique designed to enhance seed germination, particularly under stressful environmental conditions [81]. By treating seeds prior to sowing, growers can enhance germination uniformity and imprint stress tolerance onto the developing seedling, a concept known as priming memory (Figure 2). Recent studies have quantified the significant advantages of this method using plant-based biostimulants. For instance, priming bitter gourd (*Momordica charantia*) seeds with a 5% Moringa leaf extract was found to be the optimal concentration, increasing the final germination percentage to 89.95%, a plant height of 26.55 cm and chlorophyll content of 41.73% [82].

Furthermore, nanotechnology is enhancing these outcomes: priming lentil seeds with chitosan nanoparticles resulted in a 12.6% increase in germination potential, a 65.9% increase in root length, a 97.4% increase in shoot length, a 73% increase in total seedling length, a 19.7% rise in seedling dry weight. Additionally, hydrolytic enzyme activities, including amylase, protease, dehydrogenase and phytase, were substantially elevated in primed seeds [83].

The effectiveness of plant extracts is significantly determined by their concentration, as excessive dosages may inhibit plant growth; research suggests that foliar sprays at concentrations of ≤0.05 (*v*/*v*) are optimal for balancing disease control with yield improvement [76]. While foliar application is often preferred for its immediate absorption and ability to bypass soil adsorption issues, soil drenching has proven superior in specific scenarios, such as enhancing drought resistance in *Spiraea* and *Pittosporum* [84]. Complementing these methods, seed priming has emerged as a vital pre-sowing strategy where extracts are used to activate metabolic processes, thereby improving germination uniformity and seedling vigor under stress conditions [85]. The ultimate quality of the extract relies fundamentally on the initial plant part used, the extraction solvent, and the methodology employed.

**Figure 3:**
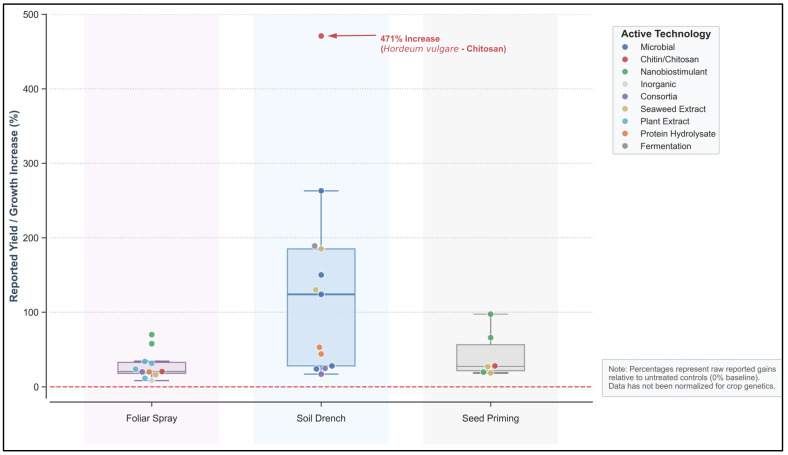
Comparative distribution of biostimulant efficacy across primary application pathways. Box plots depict the median and interquartile ranges of reported yield and physical growth increases, while the overlaid swarm plot illustrates individual study outcomes categorized by active technology. The red dashed line establishes the untreated control baseline (0% increase).

The current reliance on distinct application methods masks significant physiological and operational trade-offs that limit field-scale reliability (Figure 3). Foliar spraying, while lauded for rapid nutrient delivery, is inherently hampered by cuticular hydrophobic barriers and high susceptibility to stochastic environmental losses like rain-induced washout [14]. Conversely, soil drenching, despite its potential for sustained rhizosphere modulation, frequently suffers from inconsistent efficacy due to soil-chemical buffering and complex microbial competition that often mask the biostimulant’s impact. Seed priming, while cost-efficient for early vigor, remains operationally fragile, as its priming memory is highly dose-sensitive and often dissipates rapidly, failing to provide protection against late-stage abiotic stresses. These limitations reveal that the field lacks a standardized framework for method selection [14].

## 6. Mechanism

Understanding how plant extract-based biostimulants function requires looking beyond general growth effects to the specific molecular and physiological pathways they trigger. These extracts act as complex metabolic enhancers that influence plant physiology through three primary modes: hormonal modulation, metabolic upregulation, and transcriptional/epigenetic regulation [86].

### 6.1. Hormonal Modulation and Signaling

#### 6.1.1. Auxin-like Activity

Humic substances and PHs exhibit auxin-like effects, often attributed to the presence of indole-3-acetic acid (IAA) or precursors like tryptophan [13]. Trevisan et al. [87] demonstrated that HS acts as an auxin-mimetic non-microbial biostimulant containing 34 ± 0.31 nM of free IAA, which triggers the rapid upregulation of the early auxin-responsive gene *IAA19*. This induction promotes lateral root initiation via *AUX1*-dependent transport and stimulates H-ATPase transcription, facilitating the apoplast acidification and cell wall loosening necessary for enhanced root development. Additionally, research on *Arabidopsis*, tomato, and barley demonstrates that chitosan irrigation significantly alters root architecture by promoting a 3.6-fold accumulation of IAA and a 2–3 fold increase in salicylic and jasmonic acids. These hormonal shifts, occurring alongside an 8-fold induction of YUCCA2 and the repression of the stem cell regulator WOX5, result in a 2.5-fold reduction in plant biomass and an 80% decrease in secondary root formation at high doses [88].

#### 6.1.2. Cytokinin and Gibberellin Mimicry

Pizzeghello et al. [89] demonstrated that various humic substances contain the cytokinin isopentenyladenosine in physiologically active concentrations of 34–145 pmol mg^−1^ C. Specifically, humic fractions from earthworm feces showed the highest IPA content, which significantly stimulated plant metabolism by increasing chlorophyll levels up to 2.7-fold and sulfur-assimilation enzyme activity by more than 6-fold. Furthermore, Stanga et al. [90] identified that SMAX1, a member of an eight-gene family most abundant in dry seeds, acts as a specific downstream repressor that must be degraded via the MAX2-ubiquitin ligase to lift the inhibition of germination and photomorphogenesis. Mechanistically, this process restores the expression of critical signaling markers like *D14-LIKE2* and *KAR-UP F-BOX1*, effectively mimicking the wild-type response to karrikin and strigolactone treatments.

#### 6.1.3. Hormonal Homeostasis

Beyond direct mimicry, biostimulants modulate endogenous hormone levels. Wally et al. [91] observed that *Ascophyllum nodosum* extracts can downregulate auxin biosynthesis genes while simultaneously upregulating genes responsible for cytokinin and ABA biosynthesis, thereby fine-tuning the plant’s growth-defense balance.

### 6.2. Metabolic and Enzymatic Regulation

Biostimulants directly enhance the catalytic efficiency and concentration of critical enzymes, driving metabolic flux and stress defense:

#### 6.2.1. Nitrogen and Carbon Metabolism

Non-microbial Biostimulants like HS and PHs induce the activity of key nitrogen-assimilating enzymes. Ertani et al. [92] reported that organic biostimulants, specifically PHs from alfalfa and meat, and HS from sources like earthworm feces, significantly enhance plant growth by mimicking or containing phytohormones. PHs were found to exhibit gibberellin-like and weak auxin-like activities, which stimulated root architecture and accelerated nitrogen metabolism in maize by increasing Nitrate Reductase and Glutamine Synthetase activities. Similarly, HS were found to contain the cytokinin isopentenyladenosine, with the earthworm-derived fraction showing the highest hormonal activity and boosting chlorophyll, protein, and sulfur-related enzyme levels. Together, these results confirm that these substances improve plant productivity not just through nutrition, but by actively regulating hormonal signaling and metabolic pathways related to nitrogen and sulfur assimilation.

#### 6.2.2. Secondary Metabolism (Phenylpropanoid Pathway)

A widely observed mechanism is the stimulation of Phenylalanine Ammonia-Lyase (PAL) activity. Schiavon et al. [93] showed that HS enhances phenylpropanoid metabolism by inducing *PAL1* transcript accumulation. Similarly, De Saeger et al. [94] demonstrated that seaweed extracts, specifically from *Ascophyllum nodosum*, enhance plant tolerance by inducing antioxidant enzymes and altering metabolic profiles to combat stress-induced oxidative injury. Their research showed that these extracts trigger a priming effect, which includes the accumulation of defensive metabolites and the activation of defense-related genes like *MAPK* and *WRKY*s to neutralize reactive oxygen species.

#### 6.2.3. Cellular Transport

The activation of PM H-ATPase is crucial for nutrient uptake. Quaggiotti et al. [95] demonstrated that small molecular size humic substances significantly up-regulate the expression of Mha^2^ in roots, which encodes a plasma membrane H(+)-ATPase. This activity stimulates the electrochemical gradient necessary to drive active nitrate influx into the plant.

### 6.3. Transcriptional and Epigenetic Regulation

Recent advances have revealed that biostimulants function at the genomic level, altering gene expression profiles and potentially imprinting stress memory.

#### 6.3.1. Targeted Gene Expression

Biostimulants modulate the transcription of specific transporter genes. A study on maize seedlings demonstrated that an 8-fold up-regulation of the *Mha2* H+-ATPase gene in roots was accompanied by a 70% increase in nitrate influx following treatment with low molecular size humic substances [95]. Additionally, A comparative analysis of two *Ascophyllum nodosum* extracts showed that a 4-fold difference in polyphenol content corresponded to distinct gene expression profiles, with the two biostimulants dysregulating 4.47% and 0.87% of the transcriptome, respectively [96].

#### 6.3.2. Transcription Factors (TFs)

Biostimulants influence master regulators of stress and growth. Aamir et al. [97] reported that bio-priming tomato plants with *Trichoderma erinaceum* elicited a robust defense response, characterized by a 16.51-fold upregulation of *SlWRKY31* in roots and a 14.07-fold increase in *SlWRKY37* in leaves, which coincided with a 32.06% reduction in H_2_O_2_ generation.

#### 6.3.3. miRNA and Epigenetics

Emerging evidence suggests biostimulants may influence post-transcriptional regulation. Shukla et al. [98] discovered that *Ascophyllum nodosum* extract mitigates salinity stress in *Arabidopsis* by modulating the expression of specific microRNAs (miRNAs), such as miR399 and miR827, which are involved in phosphate homeostasis and stress tolerance pathways.

## 7. Effects of Biostimulants on Abiotic and Biotic Stress

Biostimulants have emerged as a pivotal technology in sustainable agriculture, distinct from traditional fertilizers and plant protection products. Unlike conventional inputs that directly target pests or supply nutrients, biostimulants operate by modifying plant physiology to enhance resilience. Recent literature (2023–2026) emphasizes their role as priming agents, capable of pre-activating plant defense systems against both abiotic (environmental) and biotic (biological) stressors [99,100] (Table 2).

### 7.1. Mitigation of Abiotic Stress

Abiotic stresses such as drought, salinity, extreme temperatures, and heavy metal toxicity are the primary drivers of yield loss globally. Biostimulants mitigate these effects by reprogramming plant metabolism to maintain growth under hostile conditions.

#### 7.1.1. Osmoregulation and Water Balance

Under drought and salinity stress, plants risk cellular dehydration. According to Di Sario et al. [99], osmocompatible solutes (OCSs) are small organic molecules that accumulate in the cytosol and vacuoles of plant cells without causing cellular damage. These solutes, which include sugar alcohols (polyols), amino acids (like proline), and betaines, help maintain cell volume and turgor pressure, thereby preventing plasmolysis and shrinkage caused by water loss under drought and salinity stress. Recent metabolomic studies revealed that biostimulants, such as microalgal extracts and protein hydrolysates, trigger a specific metabolic reprogramming under stress, enhancing the production of lipids and tricarboxylic acid cycle intermediates to stabilize membranes and sustain energy production [101]. For instance, recent studies on pea (*Pisum sativum* L.) crops subjected to water deficit, biostimulant application maintained photosynthetic activity at 82%, significantly higher than the 60% observed in untreated controls. This physiological stability was supported by a 145% increase in superoxide dismutase and catalase activities and a more robust root architecture that was 10 cm longer than controls, effectively halving the total yield loss [102].

#### 7.1.2. Antioxidant Defense Systems

Abiotic stress triggers the overproduction of Reactive Oxygen Species (ROS), which induces oxidative stress and damages cell membranes. Biostimulants mitigate this damage by enhancing the activity of antioxidant enzymes such as superoxide dismutase and catalase, as well as non-enzymatic antioxidants, including polyphenols and flavonoids. Transcriptomic analysis has confirmed that biostimulants, such as *Ascophyllum nodosum* extracts, can upregulate genes encoding for Heat Shock Proteins (HSPs) and ROS-scavenging enzymes, thereby protecting the photosynthetic apparatus from thermal and oxidative stress. Specifically, studies on amino acid-based biostimulants have demonstrated a priming effect characterized by the differential expression of 107 transcription factors, specifically the upregulation of 71 and the downregulation of 36 distinct factors involved in the orchestration of the heat stress response [103].

Recent research has highlighted the potent role of natural and seaweed-based biostimulants in fortifying plant defense mechanisms against environmental stressors. Belal et al. [104] demonstrated that foliar applications of bee honey significantly mitigated drought stress in *Phaseolus vulgaris* by reducing malondialdehyde and hydrogen peroxide levels while simultaneously boosting the activity of superoxide dismutase and catalase.

#### 7.1.3. Hormonal Modulation and Gene Priming

Biostimulants can regulate plant hormone signaling, particularly ABA, which acts as a mediator in plant resilience by controlling stomatal closure during drought. Some plant-derived biostimulants modulate ABA levels to balance transpiration with photosynthesis, for instance, in pea (*Pisum sativum* L.) crops, this optimization helped maintain photosynthetic activity at 82% compared to 60% in untreated stressed plants [99]. Validation through RNA-sequencing has shown that humic substances act as priming preconditioners, triggering a primed memory that allows plants to respond faster and more effectively to subsequent stress events. These substances upregulate genes involved in auxin, gibberellic acid, and ABA metabolism even before stress occurs, leading to morphological improvements such as enhanced root systems (up to 10 cm longer) and significant enhancements in fruit weight (up to 37%) and shoot fresh weight (up to 52%) under deficit irrigation [99,103].

### 7.2. Mitigation of Biotic Stress

Although biostimulants act differently from pesticides and lack direct biocidal activity, they are instrumental in fortifying plant immunity through the modulation of indirect physiological mechanisms.

#### 7.2.1. Priming and Induced Resistance

The primary mechanism for stress mitigation involves the use of microbial biostimulants, such as PGPR and AMF, to enhance environmental stress tolerance by activating genes responsible for the antioxidant defense system. These biostimulants put the plant in a state of alert through Induced Systemic Resistance (ISR), a phenomenon specifically noted with the use of beneficial microorganisms like *Debaryomyces hansenii*. Recent reviews in this issue highlight that these microbial agents trigger the solubilization of minerals and the production of phytohormones, secondary metabolites, and volatile organic compounds to effectively enhance plant health and productivity [100].

#### 7.2.2. Physical and Chemical Barriers

Biostimulants enhance physical and chemical barriers by strengthening the plant’s structural integrity. While silicon is a primary example, recent classifications of ‘beneficial elements’ in biostimulant research have expanded to include Selenium, Cobalt, and Sodium. These elements provide constitutive structural reinforcement or activate transient stress-response pathways, ensuring physiological stability under environmental pressure [103]. These treatments further fortify plants by increasing the production of secondary metabolites, such as phenols and flavonoids, which serve as biochemical barriers against pathogens and herbivores. By promoting this structural reinforcement and accumulating antioxidant metabolites, biostimulants help maintain membrane stability and ensure physiological resilience under environmental pressure [105].

#### 7.2.3. Rhizosphere Competence

Rhizosphere competence is the ability of beneficial microbes like *Bacillus* and *Paenibacillus* sp. to colonize the root-adjacent soil, often forming biofilms that physically shield plants from soil-borne pathogens. These competent microbes improve crop resilience by enhancing nutrient uptake and triggering systemic acquired resistance (SAR), which stimulates the production of antioxidants like phenols and flavonoids to combat abiotic stresses such as drought and salinity. By establishing a stable presence in the rhizosphere, these biostimulants also modulate enzymatic activities, such as polygalacturonase and pectin methylesterase, to reduce oxidative stress and extend fruit shelf life [26].

#### 7.2.4. Impact on Crop Quality Traits (Omics Evidence)

**Figure 4:**
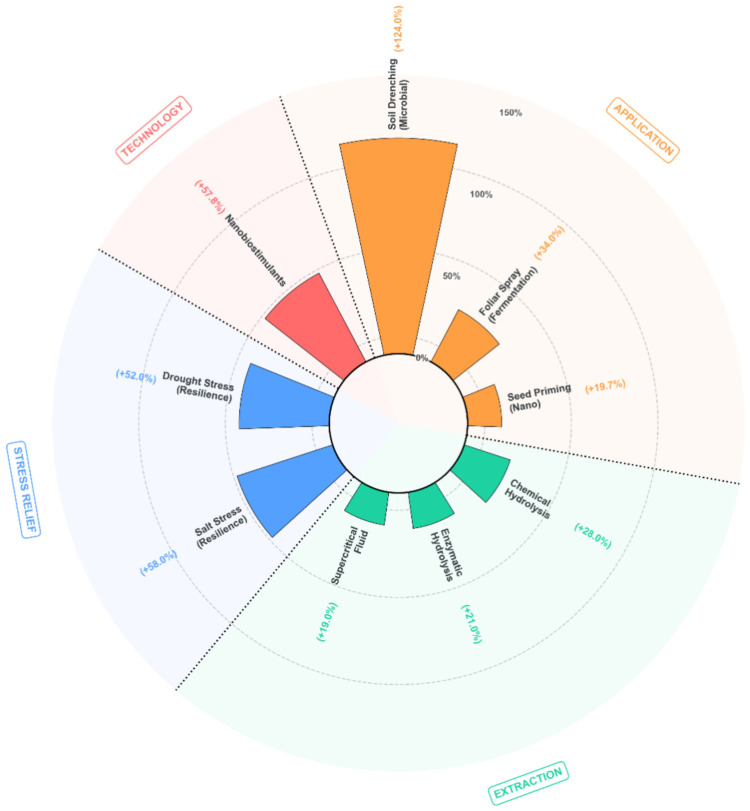
Comparative analysis of agricultural biostimulant advancements categorized by Application Method, Extraction Process, Technology Type, and Stress Resilience Impact. Percentages indicate the relative growth or efficacy.

Beyond defense, advanced proteomic studies have linked biostimulant application directly to processing quality. Research on wheat has demonstrated that specific biostimulants can alter the gliadin/glutenin ratio via post-translational modifications, improving the glutenin-to-gliadin ratio by approximately 7%, improving dough rheology and bread-making quality, proving that these agents influence complex quality traits alongside stress resilience [103]. The overall comparative performance of all processes is shown in Figure 4.

**Table 2:** Comprehensive summary of non-microbial biostimulant feedstocks, processing routes, bioactive compounds, crop responses, application modes, and major limitations.

Feedstock	Extraction	Bioactive Compound	Crop Response	Application Mode	Limitation	Reference
Brown algae (e.g., *Ascophyllum nodosum*, *Fucus* spp.)	Classical (hot water, acid, salt) and Modern (enzyme-assisted, microwave)	Fucoidan	Enhanced abiotic stress tolerance (cold, drought, salinity) and pathogen defense priming	Exogenous bio-fertilizer/Seedling treatment/Post-harvest fruit coating	Extraction conditions heavily alter structural integrity; lacks extensive research on potential negative plant effects	[106]
*Linum usitatissimum* L. (flaxseed)	Solid–liquid hot water extraction (infusion)	Micro/macro-nutrients (K, Zn), amino acids (glutamic acid), fatty acids (oleic/elaidic), carbohydrates (sucrose)	Increased plant height, pod count, and seed yield; modified seed fatty/amino acid profiles	Foliar spray (vegetative and flowering stages)	Slight reduction in 1000 seed weight/total protein; precise application timing required	[107]
Agri-food waste (pasta by-products/PHA-rich bacterial biomass)	Mild chemical (alkaline/acid) and enzymatic (alcalase/papain) hydrolysis	PHs, including free amino acids and peptides	Enhanced root elongation; increased overall shoot and root biomass	Seed priming, foliar spray, and soil drench	Multi-step bacterial fermentation increases production costs and carbon footprint compared to direct plant hydrolysis	[108]
Spirulina *(Arthrospira platensis)* residual biomass	Ultrasound-assisted extraction (UAE) waste stream	Free amino acids (e.g., lysine, phenylalanine) and peptides	Enhanced Soil Organic Carbon (SOC) sequestration; regeneration of degraded soils	Soil application (precision agricultural drones)	Long-term SOC permanence is uncertain; carbon-negative potential heavily depends on the product-to-waste ratio	[109]
*Salvia rosmarinus*	Soxhlet extraction (Ethanol)	Carnosic acid, phenolic compounds	root/seedling growth	Foliar/Seed priming (formulated with carriers)	Dual-metabolite behavior (potential phytotoxicity at high doses); dose-dependent optimization required	[21]
*Rosmarinus officinalis* (Distillation water residue)	Hydrodistillation (byproduct)	Rosmarinic acid, polyphenols	Enhanced shoot height, total biomass, chlorophyll content	Foliar spray	Dose-dependent phytotoxicity; requires optimization of concentration	[110]

## 8. Regulations

Driven by the EU Nitrates Directive and the Farm to Fork Strategy’s mandate to reduce nutrient losses and fertilizer dependence, the regulatory landscape for plant biostimulants underwent a paradigm shift with the full implementation of Regulation (EU) 2019/1009 (EU FPR) on 16 July 2022 [111]. This framework replaces fragmented national schemes with a unified system, facilitating the free movement of CE-marked products across member states.

Crucially, the FPR defines biostimulants by function rather than composition: products must stimulate plant nutrition processes independently of their nutrient content. This strictly distinguishes them from conventional fertilizers. To achieve compliance, a product must scientifically demonstrate efficacy in improving NUE, abiotic stress tolerance, quality traits, or the availability of confined soil nutrients. This regulation supports the production of biostimulants derived from organic and waste materials within the EU, promoting the sustainable application of nutrients [111]. Plant biostimulants are categorized under PFC 6, microbial biostimulants (PFC 6(A)) and non-microbial biostimulants (PFC 6(B)). The regulation subjects biocompounds to specific safety regulations, including stringent limits on contaminants. These contaminants encompass heavy metals and must also meet pathogen limits, ensuring the absence of harmful bacteria such as *Salmonella* spp. and *Escherichia coli* [111].

**Figure 5:**
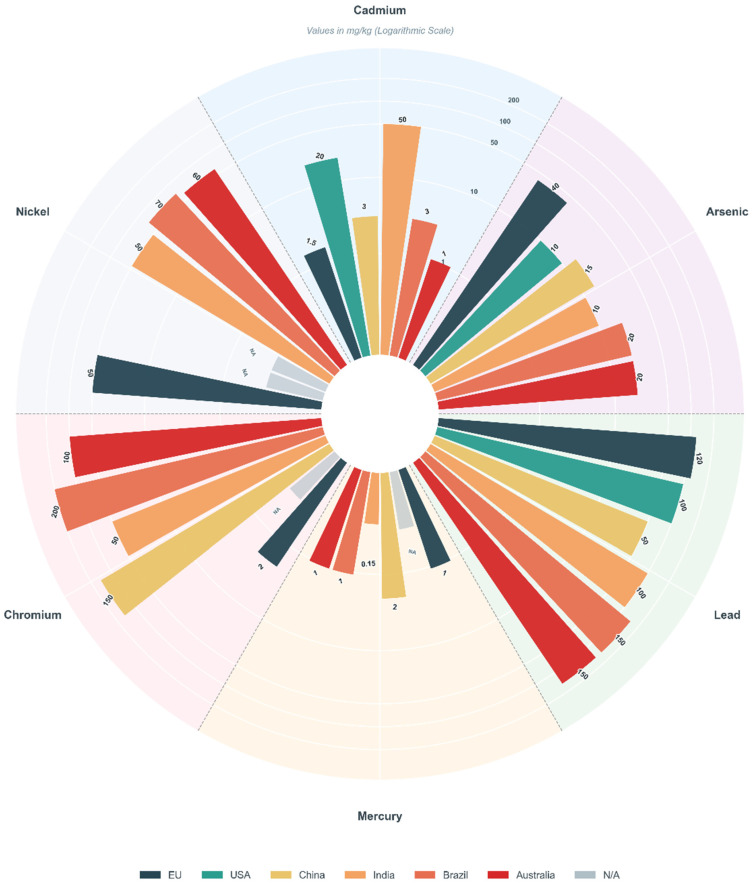
Comparative regulatory limits for heavy metal contaminants in fertilizers and soil improvers across six major regions (EU, USA, China, India, Brazil, and Australia). Values are expressed in mg/kg on a logarithmic scale. “NA” indicates that the specific contaminant is unregulated or no limit was defined for that jurisdiction.

Outside the EU, regulatory approaches to plant biostimulants vary considerably across major agricultural economies. In the United States, there is currently no statutory federal definition of plant biostimulants, and regulation remains largely claims-based: products making pesticidal or growth-regulating claims fall under the Federal Insecticide, Fungicide, and Rodenticide Act (FIFRA) and require EPA registration, whereas products claiming benefits related to NUE, abiotic stress tolerance, or soil quality are generally exempt and regulated at the state level, with the USDA playing a research and advisory role [112]. In contrast, India has established a dedicated national framework through amendments to the Fertilizer Control Order (FCO), 1985, ref. [113] formally recognizing biostimulants as a distinct category and requiring centralized registration supported by efficacy and safety data under the Ministry of Agriculture and Farmers’ Welfare (Figure 5). China, meanwhile, regulates biostimulant-type products within existing fertilizer and soil amendment regulations overseen by the Ministry of Agriculture and Rural Affairs (MARA), without a standalone biostimulant definition; products are classified according to composition (e.g., amino acids, humic substances, microbial formulations), resulting in a structured but non-harmonized system. Together, these contrasting approaches highlight the global regulatory heterogeneity surrounding biostimulants, which has important implications for innovation, market access, and international trade [114].

- United States (Pathogens): While there is no federal biostimulant law, most states align with EPA Part 503 (Class A Biosolids) standards for pathogens. This requires *Salmonella* to be effectively absent (<3 MPN per 4 g) and Fecal Coliforms to be below 1000 MPN/g [112].
- China (Microbial Limits): The standard GB 20287-2006 for microbial inoculants is stricter than many others regarding coliforms, requiring Fecal Coliforms to be <100 CFU/g (compared to the EU/US limit of 1000) [114].
- Australia: Australia often has the strictest testing protocols. Under AS 4454, *Salmonella* must be absent in a larger sample size (50 g vs. the standard 25 g) to ensure higher safety confidence [115].
- Brazil (Helminths): Uniquely, Brazil’s IN 61/2020 also explicitly requires testing for Viable Helminth Eggs (must be <1 per 4 g), addressing risks specific to tropical climates that other regions might not prioritize in their primary biostimulant text [116].

While organic carbon is typically classified as a soil conditioner rather than a direct metabolic enhancer, it plays a critical regulatory role in Asian markets, specifically India and China. In these regions, regulatory frameworks utilize Organic Carbon content as a mandatory quality floor to verify the authenticity of biological products.

Unlike the EU or US, which focus primarily on efficacy claims, India’s Fertilizer Control Order (FCO) and China’s National Standards enforce minimum Organic Carbon percentages (e.g., ≥14% in India for specific categories) to serve as a proxy for biological origin [114]. This composition-based approach effectively acts as an anti-counterfeiting measure, preventing the market entry of ambiguous biostimulants, such as synthetic chemical salts mixed with dyes by ensuring that the product contains a genuine baseline of biological matrix derived from raw materials like leonardite, peat, or seaweed (Table 3).

## 9. Artificial Intelligence and Machine Learning in Biostimulants

The transition into the “Biostimulants 4.0” era represents a fundamental paradigm shift where the industry moves beyond empirical trial-and-error testing towards a predictive, systems-biology approach driven by AI and Machine Learning (ML). While the integration of AI is highly promising, moving from theoretical models to field-ready applications requires addressing critical computational constraints [117].

The efficacy of predictive ML models in agriculture is fundamentally dependent on dataset size, quality and architecture. Linear regression models and their extensions, such as Ridge, Lasso, and Elastic Net, are effective for predicting crop performance from climatic variables while mitigating overfitting in complex datasets. However, for non-linear relationships, advanced models are preferred for their ability to handle high-dimensional, heterogeneous agricultural data. Specifically, Support Vector Regression (SVR) employs kernel functions to map non-linear data into higher-dimensional feature spaces, proving valuable for predicting metrics like water consumption and soil temperature. Meanwhile, ensemble methods such as Random Forest (RF) and eXtreme Gradient Boosting (XGBoost) leverage decision-tree architectures and gradient-boosting to handle large, noisy datasets with high robustness [117]. Recent industry-academic collaborations demonstrate this transition; AI platforms are now actively utilized to analyze massive proteomics and metabolomics datasets to pinpoint specific cellular biomarkers, which accurately predict how plants will respond to abiotic stressors following biostimulant application. For instance, Lucini et al. [118] utilized metabolomic profiling to demonstrate that vegetal biopolymer-based non-microbial biostimulants could trigger a 56% increase in melon root biomass by specifically modulating brassinosteroid signaling and stress-related metabolic pathways. Similarly, Lephatsi et al. [119] employed untargeted metabolomics and AI-driven clustering to reveal how *Bacillus*-based formulations induce metabolic reprogramming and upregulate drought-response genes in maize, effectively priming the plant for environmental stress.

Beyond initial biomarker discovery, advanced ML models, particularly, Support Vector Regression (SVR), Random Forests (RF), and eXtreme Gradient Boosting (XGBoost) are increasingly preferred for their capacity to handle high-dimensional, heterogeneous agricultural data and predict complex crop traits linked to biostimulant applications [117]. The reliability of these models is typically assessed through standard statistical performance metrics, primarily the Coefficient of Determination (R^2^), which quantifies the model’s explanatory power, and the Root Mean Square Error (RMSE) or Mean Absolute Error (MAE), which evaluate the deviation between predicted biostimulant efficacy and actual field outcomes [117]. These models have now achieved up to 87% accuracy in predicting the efficacy of microbial strains for drought mitigation. However, reliable AI performance is constrained by dataset requirements; models are prone to ‘data hunger’ and poor generalizability if not trained on high-quality, longitudinal multi-omics and environmental metadata. Furthermore, validation must extend beyond basic training-testing splits, employing k-fold cross-validation or nested resampling to ensure predictive power remains consistent across diverse geographic and seasonal conditions.

By integrating these predictive analytics with Precision Agriculture tools, such as IoT-enabled digital twins and computer-vision sprayers that can reduce input waste by 70–95%, the Biostimulants 4.0 era ensures that biological treatments are no longer “one-size-fits-all” but are instead site-specific, genotype-targeted, and ecologically optimized solutions.

Despite these advancements, significant hurdles remain. A major limitation is generalizability; algorithms often suffer performance degradation when applied to different genotypes or open-field environments. Critically, a bottleneck remains in quantifying prediction uncertainty, as models often lack frameworks to account for the stochastic nature of rainfed systems, and in the practical implementation of ‘black-box’ architectures. This lack of transparency introduces an adoption barrier, as end-users require algorithms that elucidate the reason behind a predicted interaction [119]. Consequently, the integration of Explainable AI (XAI) frameworks, such as SHapley Additive exPlanations (SHAP) and Local Interpretable Model-agnostic Explanations (LIME), is mandatory [120]. Future research must prioritize these XAI frameworks alongside the standardization of input metadata to facilitate the development of more transferable, robust models [121]. Finally, actual field deployment requires overcoming infrastructure bottlenecks, including the need for reliable rural broadband, edge-computing capabilities, and durable sensor networks [119].

## 10. Environmental and Sustainable Impacts

The integration of biostimulants into modern agricultural practices is a cornerstone of sustainable intensification, the goal of increasing food production while simultaneously reducing the environmental footprint of farming. Beyond mere yield enhancement, the recent literature emphasizes the role of biostimulants in mitigating climate change, improving resource efficiency, and ensuring economic profitability for growers.

### 10.1. Nutrient Use Efficiency and Reduced Environmental Losses

A primary environmental benefit of biostimulants is their ability to enhance NUE, allowing for reduced application of synthetic fertilizers without compromising yield. Rouphael and Colla [122] emphasize that both microbial and non-microbial biostimulants improve the uptake and assimilation of macro-nutrients, which is critical for reducing the environmental load of agriculture. Giordano et al. [123] reported that specific PH biostimulants increased NUE by up to 25% in leafy vegetables. This efficiency allows farmers to reduce synthetic nitrogen application rates, directly mitigating the release of nitrous oxide (N_2_O), a greenhouse gas with 298 times the warming potential of CO_2_. By upregulating nitrate transporter genes (e.g., *NRT1.1*) and stimulating root proliferation (increasing root biomass by 9.3% to 46.6% in lettuce treatments), biostimulants significantly minimize nitrate leaching into groundwater, addressing a critical eutrophication risk.

### 10.2. Life Cycle Assessment (LCA): Quantifying the Footprint

To validate these environmental benefits scientifically, researchers are increasingly applying LCA methodologies to track their impact from production to field application (“cradle-to-grave”). The primary driver of decarbonization is the avoidance of the Haber-Bosch process used for synthetic nitrogen, which consumes vast amounts of natural gas. Mulya et al. [124] validated this mechanism, calculating that the production of microbial bio-based fertilizers generates 23.2 times less carbon emissions than equivalent synthetic nitrogen fertilizers. By shifting to bio-based inputs, the industry effectively eliminates the massive, embedded carbon cost of crop nutrition.

Additionally, the integration of biostimulants into Carbon Farming represents a shift from traditional input-output models to a Decarbonization Finance framework. While agriculture generates nearly one-third of global greenhouse gas (GHG) emissions, it currently receives less than 5% of global climate finance. Biostimulants act as dual-action tools for emissions avoidance and carbon removal, making them essential for closing this financing gap [125]. The Gold Standard (2023) “SOC Activity Module for Biostimulants” now provides a formal framework to quantify GHG changes from soil revitalization [126]. This methodology specifically recognizes that biostimulants activate the soil microorganisms required for net carbon sequestration [126]. Furthermore, the voluntary carbon market is projected to reach $35 billion by 2030.

High-quality agricultural credits that sequester soil carbon currently command a premium, priced between $10 and $35 per tonne, significantly higher than the $6.50 average for lower-integrity generic credits. The EU Carbon Removals and Carbon Farming (CRCF) Regulation (2024) has established the first EU-wide voluntary system for certifying soil emission reductions, allowing farmers to commercialize carbon reduction as a secondary product [127]. By leveraging these markets, farmers can transform biostimulant use into a yield insurance policy that pays direct dividends through Verified Carbon Units (VCUs).

While these decarbonization frameworks offer a promising economic incentive for biostimulant adoption, the conversion of ‘emissions avoidance’ into verifiable revenue requires a rigorous evidence-based approach. Current claims regarding soil sequestration potential and economic value generation remain susceptible to over-estimation in the absence of site-specific data [7]. To achieve commercial and regulatory credibility, industry claims must move beyond qualitative benefits toward integrated quantitative validation. This entails the systematic use of LCA to quantify net carbon footprints, accounting for emissions from feedstock sourcing and processing, and Techno-Economic Analysis (TEA) to define the operational cost–benefit thresholds required for carbon credit eligibility [7]. As mentioned in Section 4.3, the environmental performance of these inputs is a function of the entire lifecycle; therefore, future carbon farming strategies must rely on data-driven models that verify sequestration outcomes against these standardized LCA and TEA benchmarks to ensure authentic decarbonization finance.

### 10.3. Farmer’s Perspective and Socio-Economic Barriers

The successful transition from Greenhouse to Field depends not only on biological efficacy but also on the behavioral and economic realities of agricultural producers. While biostimulants are a cornerstone of Agriculture 4.0, their adoption is often slowed by a complex interplay of individual, social, and structural factors. Research conducted in previous years identifies several recurring hurdles that prevent biostimulants from becoming a universal standard in crop nutrition.

High upfront costs are the most significant barrier, particularly for small-scale and marginal farmers who operate on thin profit margins. Even when long-term ROI is positive, the initial input shock can be prohibitive. Additionally, lack of technical knowledge regarding proper application timing and dosage often leads to inconsistent results. Skepticism remains high among farmers who are accustomed to the immediate visible effect of synthetic fertilizers, whereas biostimulant results may be delayed or primarily visible under stress conditions. Furthermore, the lack of standardized quality floors (except in specific markets like EU) makes it difficult for farmers to distinguish between high-quality products and non-reliable biostimulants.

### 10.4. Food Safety and Consumer Health

Biostimulants contribute directly to public health by improving the nutritional quality of produce and reducing harmful accumulation. Excess nitrates in leafy greens (like rocket and spinach) are a significant safety concern. Colla et al. [22] and Di Mola et al. [49] found that plant-based biostimulants (specifically protein hydrolysates) stimulate the activity of nitrate reductase, the enzyme responsible for converting nitrates into amino acids. This process ensures that nitrogen is used for growth rather than stored as potentially toxic nitrates, keeping levels well within legal limits [49]. Additionally, Biostimulants act as metabolic elicitors. Rouphael et al. [122] confirmed that these products increase the production of secondary metabolites, such as total phenols, flavonoids, and Vitamin C (ascorbic acid). For instance, in greenhouse trials, biostimulant application increased total ascorbic acid content by nearly 95% in certain leafy crops [49].

### 10.5. Soil Health and Biodiversity

A critical but often overlooked aspect is the impact on soil biology. Unlike some chemical inputs that may degrade soil microbial diversity, biostimulants, particularly microbial inoculants and humic substances, act as biological enhancers. In contaminated soils, biostimulants help mitigate the uptake of toxic elements. Bartucca et al. [128] review how biostimulants improve antioxidant defense systems and modulate metal transporters, reducing the translocation of heavy metals like cadmium (Cd) and lead (Pb) into the edible parts of the crop.

## 11. Challenges and Limitations

Despite the rapid advancement of the “Biostimulants 4.0” paradigm, several fundamental limitations currently restrict the sector’s scalable deployment and the interpretability of existing literature.

- Unlike synthetic inputs or standardized virgin raw materials, agro-industrial and food waste streams are inherently variable, showing immense batch-to-batch heterogeneity driven by seasonal changes, geographic origin, and shifting supply chain conditions. This unpredictability complicates the industrial standardization of bioactive profiles. Furthermore, a critical bottleneck in waste valorization is the risk of contaminant concentration; processing agricultural residues can inadvertently concentrate heavy metals, persistent pesticide residues, or biological pathogens present in the raw waste. Overcoming these chemical and biological safety hazards necessitates intensive pre-treatment, purification, and quality-testing stages, which frequently increase production costs and elevate the overall energy and chemical footprint of the extraction process, potentially undermining the net sustainability benefits of the circular bioeconomy loop.
- Unlike synthetic fertilizers, biostimulants are complex mixtures lacking harmonized global standards for quality control. This regulatory void results in severe data heterogeneity across studies, which often employ disparate extraction metrics and evaluation criteria, preventing robust meta-analyses. Consequently, there is a critical need to shift from phenomenological observations toward understanding precise physiological mechanisms of action [129].
- Products that demonstrate significant biomass increases in controlled environments frequently exhibit inconsistent efficacy in open fields due to fluctuating temperatures, rainfall, and soil heterogeneity. Crucially, the reported effects of biostimulants depend strongly on the specific crop species, cultivation conditions, and application methods employed, making direct quantitative comparisons among studies inherently difficult. Furthermore, biostimulant responses are highly context-dependent, often showing benefits only under specific abiotic stresses, and vary drastically between cultivars, creating a pressing need for tailored, crop-specific formulations.
- Maintaining the shelf-life and field viability of microbial biostimulants is technically demanding due to their susceptibility to UV, heat, and incompatible tank mixes. Similarly, advanced nanobiostimulants face their own greenhouse-to-field gap; they frequently aggregate or degrade prematurely in complex soil matrices. Their unknown environmental fate, potential ecotoxicity, and high synthesis costs currently limit their adoption primarily to high-value horticulture.
- The theoretical promise of AI and nanobiostimulants is hindered by a severe lack of open-field, commercial validation. The current literature reflects a geographic and economic bias toward the Global North and high-value crops, largely ignoring broad-acre agriculture in developing economies [121]. Furthermore, the reliance on single-season studies leaves the multi-year ecological impacts on native soil microbiomes as a critical scientific blind spot [130].
- A major research gap exists in the absence of globally harmonized regulatory standards for waste-derived inputs. The current fragmented landscape, where definitions and quality requirements fluctuate between regions, significantly hinders international trade and market access for SMEs. Furthermore, commercialization is frequently blocked by a knowledge-action gap, where the economic benefits of biostimulants are not effectively communicated to farmers, and the lack of standardized, region-specific efficacy data discourages long-term investment. Future research must prioritize the development of cross-jurisdictional regulatory frameworks and robust, long-term field validation studies that provide clear Return on Investment (ROI) data to bridge the gap between technological potential and commercial adoption.

## 12. Conclusions and Future Works

The transition toward the “Biostimulants 4.0” framework represents a critical shift to the data-driven modulation of plant physiology, yielding profound agronomic and economic implications. Among our main findings, upcycling agro-industrial waste into nanobiostimulants not only overcomes traditional bioavailability constraints to enhance crop stress resilience but also generates up to 23.2 times fewer carbon emissions than synthetic nitrogen production. Furthermore, by deploying ML alongside multi-omics data, the industry is moving beyond empirical testing to precisely predict biostimulant efficacy for site-specific applications. Ultimately, the implications of these technological advancements offer a scalable solution to decarbonize intensive agriculture, providing a novel financial pathway that allows farmers to monetize improved nutrient-use efficiency through high-integrity soil carbon credits.

To fully realize the potential of sustainable agricultural intensification, future research must overcome current scalability and mechanistic limitations. The field must shift from phenomenological observations to a molecular-level understanding using advanced Omics technologies to satisfy regulatory requirements and define exact application windows. Simultaneously, product development must evolve from simple single-strain inoculants toward engineered synthetic microbial consortia that offer complementary agronomic traits. Utilizing green synthesis, researchers must also focus on developing stimuli-responsive nanocarriers that release active ingredients only in response to specific environmental triggers, such as pH shifts or pathogen presence. Ultimately, the successful commercial deployment of these advanced formulations relies on coupling them with precision agriculture and IoT sensor networks to trigger real-time, site-specific applications that maximize resource efficiency and minimize ecological risk.

Finally, as the industry moves toward widespread commercialization of waste-derived biostimulants, future research must rigorously address potential consumer safety risks, such as the carryover of allergens from feedstocks like soybean, fish, or crustacean-derived chitin. Future studies should prioritize the development of standardized purification protocols and analytical monitoring techniques to ensure that no residual allergenic peptides persist in the edible parts of the crop, thereby guaranteeing that circular bio-based inputs meet the highest safety standards for the global food supply chain. Future priorities must therefore shift toward establishing standardized, globally harmonized regulatory protocols, implementing long-term multi-year field validation studies to prove commercial viability, and creating transparent economic frameworks that simplify the path to market for high-quality, waste-derived biological products.

## Figures and Tables

**Table 3:** Comparative regulatory standards for key biostimulant active ingredients and microbial specifications across India, China, the EU, and the USA [113,114,115,116].

Active Ingredient	India (Mandatory Minimums) [FCO S-VI]	China (Mandatory Minimums) [GB/NY Stds]	EU [Reg 2019/1009]	United States [State Laws]
Humic Acids	≥6.0% (Liquid), ≥1.5% (Granules)	≥3.0% (Mineral source), ≥30 g/L (Liquid)	No fixed %, (Must declare actual content)	No fixed %, (Must declare and verify)
Fulvic Acids	≥1.0% (if claimed)	≥3.0%	No fixed %	No fixed %
Amino Acids	≥10% (Total), ≥5% (Free Amino Acids)	≥100 g/L (Liquid), ≥10% (Solid)	No fixed %, (Must declare Free Amino Acids)	No fixed %. (Often need >0.1% to list)
Seaweed Extract	>0.1% Active (varies by formulation)	≥10% (Organic matter), ≥2% (Alginic Acid)	No fixed %	No fixed %
Organic Carbon	≥14% (for some categories)	≥50 g/L	>3% (for Organic Fertilizers)	--
Chitosan (Biopolymer)	Matches Label Claim (Classified as “Biopolymer” under FCO Sch VI)	≥30 g/L (Liquid), ≥5.0% (Powder), (Standard NY/T 788 [114])	No fixed % (Regulated as Basic Substance or CMC 14)	Matches Label Claim, (Must verify % Chitosan)
Silicon (Inorganic)	Not a Biostimulant (Regulated as “Silicon Fertilizer” or “Beneficial Element”)	≥50% (SiO_2_ for conditioners), ≥100 g/L (Liquid Si fert)	Not a Biostimulant (Regulated as PFC 1: Inorganic Fertilizer)	Matches Label Claim, (Regulated as “Beneficial Substance”. Must measure Soluble Silicon)
Phosphites (Inorganic PO_3_)	Banned as Biostimulant (Strictly regulated as Fungicide or Fertilizer)	Regulated as Fungicide (Not a standard biostimulant)	Banned as Biostimulant (Prohibited in PFC 6 due to fungicidal activity)	Matches Label Claim, (Sold as Fertilizer 0-60-0, but under scrutiny)
Allowed Species	Positive List, Specific standards for *Rhizobium*, *PSB*, *KSB*, *Zinc Solubilizers*, etc.	Open List, Allows *Bacillus*, *Trichoderma*, *Pseudomonas*, Yeasts, etc.	Strictly Limited (4 Genera), *Azotobacter*, *Azospirillum*, *Rhizobium*, Mycorrhizae	Open List Any non-pathogenic species
Minimum CFU *(Solid)*	≥5.0 × 10^7^ CFU/g, (Carrier-based)	≥2.0 × 10^8^ CFU/g, (Extremely High)	No Fixed Minimum (Must match label)	No Fixed Minimum, (Must match label)
Minimum CFU *(Liquid)*	≥1.0 × 10^8^ CFU/mL	≥2.0 × 10^8^ CFU/mL	No Fixed Minimum	No Fixed Minimum
Contaminants *(Pathogens)*	Total Contamination, <10^5^ CFU/g, (Unique “Purity” Rule)	Strict Limits Fecal Coliforms: < 100 CFU/g	Strict Limits *Salmonella*: Absent, *E. coli*: <1000 CFU/g	State Limits Salmonella: <3 MPN/4 g, (EPA 503 Standard [113])
Viability	Strictly Enforced Expiry date is mandatory (usually 6–12 months).	Strictly Enforced Random market testing is common.	Stability data must be proven for shelf-life.	Truth in Labeling, claims and original numbers should be same
*Salmonella* spp.	Absent in 25 g	Absent in 25 g	Absent in 25 g	<3 MPN/4 g
*E. coli*/Coliforms	Absent in 1 g	<100 CFU/g	<1000 CFU/g	<1000 MPN/g

## Data Availability

No new data were created or analyzed in this study.

## Abbreviations

The following abbreviations are used in this manuscript:

ABA	Abscisic acid
AI	Artificial Intelligence
AMF	Arbuscular mycorrhizal fungi
CAGR	Compound annual growth rate
CBD	Cannabidiol
CFU	Colony-forming units
CRCF	Carbon Removals and Carbon Farming
EH	Enzymatic hydrolysis
EPA	Environmental Protection Agency
EU	European Union
FCO	Fertilizer Control Order
FIFRA	Federal Insecticide, Fungicide, and Rodenticide Act
FLW	Food loss and waste
FPH	Fish protein hydrolysates
FPR	Fertilizing Products Regulation
GHG	Greenhouse gas
HA	Humic acids
HCl	Hydrochloric Acid
HEf	Humic acid fraction
HSPs	Heat Shock Proteins
IAA	Indole-3-acetic acid
IoT	Internet of Things
ISR	Induced Systemic Resistance
KSB	Potassium Solubilizing Bacteria
LCA	Life Cycle Assessment
LIME	Local Interpretable Model-agnostic Explanations
MAE	Microwave Assisted Extraction
MAPK	Mitogen-activated protein kinase
MARA	Ministry of Agriculture and Rural Affairs
MENA	Middle East and North Africa
miRNA	microRNAs
ML	Machine Learning
MPN	Most Probable Number
MT	Million tons
NPs	Nanoparticles
NUE	Nutrient use efficiency
OCSs	Osmocompatible solutes
OSE	Organic solvent extraction
PAL	Phenylalanine Ammonia-Lyase
PFC	Product Function Category
PGPR	Plant-Growth-Promoting Rhizobacteria
PHA	Polyhydroxyalkanoates
PHs	Protein hydrolysates
PM	Plasma membrane
PSB	Phosphate-Solubilizing Bacteria
RF	Random Forests
ROI	Return on Investment
ROS	Reactive Oxygen Species
SAR	Systemic acquired resistance
SBE	Boiling extraction
SDG2	Sustainable Development Goal 2
SFE	Supercritical Fluid Extraction
SHAP	SHapley Additive exPlanations
SmF	Submerged fermentation
SOC	Soil Organic Carbon
SSF	Solid-state fermentation
SVR	Support Vector Regression
TFs	Transcription Factors
THC	Tetrahydrocannabinol
TiO_2_	Titanium dioxide
UAE	Ultrasound-assisted extraction
UN	United Nations
USD	United States Dollar
USDA	United States Department of Agriculture
UV	Ultraviolet
VCUs	Verified Carbon Units
XAI	Explainable AI
XGBoost	eXtreme Gradient Boosting

## Appendix A. Methodology: Literature Search and Selection Criteria

To ensure a comprehensive and reproducible synthesis of the literature regarding the intersection of circular bioeconomy, nanobiostimulants, and AI, a structured literature review was conducted.

- Database Sources: The primary literature search was performed across major scientific databases, including Scopus, Web of Science, Sciencedirect, and Google Scholar.
- Search Strings and Keywords: The search strategy utilized combinations of the following Boolean operators and keywords: (“Biostimulants” OR “Nanobiostimulants”) and (“Agro-industrial waste” OR “Waste valorization” OR “Circular economy”) and (“Artificial Intelligence” OR “Machine Learning” OR “Predictive modeling”) and (“Nutrient Use Efficiency” OR “Abiotic stress”).
- Timeframe: The search window was primarily restricted to publications from 2015 to 2026 to capture the most recent advancements in “Biostimulants 4.0,” nanotechnology, and emerging AI applications, though foundational texts defining biostimulant frameworks were included regardless of publication year.
- Inclusion Criteria: Peer-reviewed research articles, comprehensive review papers, meta-analyses, and official regulatory documents (e.g., EU FPR, FCO) published in English that directly addressed the extraction, formulation, agronomic application, or regulatory frameworks of biostimulants.
- Screening Process: Initial screening was conducted based on title and abstract relevance. Following the removal of duplicates, full-text articles were independently assessed by the authors to determine final eligibility based on the established inclusion criteria.

## References

[1] Wazeer H., Gaonkar S.S., Doria E., Pagano A., Balestrazzi A., Macovei A. (2024). Plant-Based Biostimulants for Seeds in the Context of Circular Economy and Sustainability. Plants.

[2] FAO (2025). The State of Food and Agriculture 2025: Addressing Land Degradation Across Landholding Scales.

[3] UN News (2022). UN Report: World Moving Backwards in Efforts to Eliminate Hunger and Malnutrition.

[4] Gustavsson J., Cederberg C., Sonesson U., Van Otterdijk R., Meybeck A. (2011). Global Food Losses and Food Waste-FAO Report.

[5] RMI (2024). Managing Methane Emissions in the Waste Sector.

[6] Eurostat (2025). Food Waste in the EU: 58 Million Tonnes Generated Annually.

[7] U.S. Food and Drug Administration (2024). Food Loss and Waste.

[8] Shrivastava V., Saju A., Sigurnjak I., Edayilam N., Van De Sande T., Meers E. (2026). Agronomic and environmental performance of animal manure-derived ammonium salts vs. synthetic mineral fertilisers: 4-year field trial evidence. Agric. Ecosyst. Environ..

[9] Wang S., Liu D., Sheng J. (2024). Prevention of Food Waste in China: Role and Impact of China’s Anti-Food Waste Law. Foods.

[10] World Bank (2026). Waste Management in the Middle East and North Africa.

[11] WWF (2024). Living Planet Report 2024: A System in Peril.

[12] Dhanasekaran P., Chittibabu S., Mouzeyar S., Boulaflous-Stevens A., Delattre C., Roche J. (2025). From waste to wonder: The potential of protein hydrolysates as plant biostimulants in agriculture. Bioresour. Technol. Rep..

[13] Just B.S., Díaz-Guerra L., Shrivastava V., Guerra-Gorostegui N., Llenas L., Vilaplana R., Meers E., Robles-Aguilar A.A. (2026). Effect of carbon availability, phosphorus, and water soil content on GHG emissions: Insights from a soil incubation study. Front. Soil Sci..

[14] Shrivastava V., Laasri I. (2025). Nutrient Recovery Strategies and Agronomic Performance in Circular Farming: A Comprehensive Review. Nitrogen.

[15] Quille P., Kacprzyk J., Oconnell S., Ng C.K.-Y. (2026). Plant biostimulants and their potential role in achieving the United Nations sustainable development goals. Plants People Planet.

[16] European Biostimulants Industry Council (2025). Making Competitive and Sustainable EU Agriculture a Reality: 2025 EBIC Summit Report.

[17] Jolayemi O.L., Malik A.H., Ekblad T., Fredlund K., Olsson M.E., Johansson E. (2022). Protein-Based Biostimulants to Enhance Plant Growth—State-of-the-Art and Future Direction with Sugar Beet as an Example. Agronomy.

[18] The Business Research Company (2026). Biostimulants Market Report 2026.

[19] GMI (2025). Food Waste Recycling Market Size—By Feedstock Source, Process, End Use—Global Forecast, 2025–2034.

[20] Corsi S., Ruggeri G., Zamboni A., Bhakti P., Espen L., Ferrante A., Noseda M., Varanini Z., Scarafoni A. (2022). A Bibliometric Analysis of the Scientific Literature on Biostimulants. Agronomy.

[21] de Elguea-Culebras G.O., Ferrando-Beneyto T., Melero-Bravo E., Sánchez-Vioque R. (2026). Upcycling Residues from *Salvia rosmarinus* Distillation and Agroforestry Processes into a Dual-Function Bioagrochemical with Biostimulant and Antifungal Properties. Agriculture.

[22] Colla G., Hoagland L., Ruzzi M., Cardarelli M., Bonini P., Canaguier R., Rouphael Y. (2017). Biostimulant Action of Protein Hydrolysates: Unraveling Their Effects on Plant Physiology and Microbiome. Front. Plant Sci..

[23] Arinaitwe U., Yabwalo D.N., Hangamaisho A. (2025). Unlocking the Potential of Biostimulants: A Review of Classification, Mode of Action, Formulations, Efficacy, Mechanisms, and Recommendations for Sustainable Intensification. Int. J. Plant Biol..

[24] Galindo F.S., Moreira A., Moraes L.A., Filho M.C.T., Jalal A., Oliveira C.E., Al-Babili S., Pagliari P.H. (2025). Challenges and promising opportunities for biostimulants application in sustainable agriculture. Advances in Agronomy.

[25] Vultaggio L., Ciriello M., Campana E., Bellitto P., Consentino B.B., Rouphael Y., Colla G., Mancuso F., La Bella S., Napoli S. (2024). Single or Blended Application of Non-Microbial Plant-Based Biostimulants and *Trichoderma atroviride* as a New Strategy to Enhance Greenhouse Cherry Tomato Performance. Plants.

[26] Lyu D., Backer R., Berrué F., Martinez-Farina C., Hui J.P.M., Smith D.L. (2023). Plant Growth-Promoting Rhizobacteria (PGPR) with Microbial Growth Broth Improve Biomass and Secondary Metabolite Accumulation of *Cannabis sativa* L.. J. Agric. Food Chem..

[27] Drobek M., Frąc M., Cybulska J. (2019). Plant Biostimulants: Importance of the Quality and Yield of Horticultural Crops and the Improvement of Plant Tolerance to Abiotic Stress—A Review. Agronomy.

[28] Drobek M., Cybulska J., Frąc M., Pieczywek P., Pertile G., Chibrikov V., Nosalewicz A., Feledyn-Szewczyk B., Sas-Paszt L., Zdunek A. (2024). Microbial biostimulants affect the development of pathogenic microorganisms and the quality of fresh strawberries (*Fragaria ananassa* Duch.). Sci. Hortic..

[29] López-Serrano L., Scalschi L., Simeón R., Bautista A.S., González-Hernández A.I. (2026). Harnessing the Power of Biostimulants: A Comprehensive Review of Their Role in Enhancing Agricultural Productivity and Sustainability. Appl. Sci..

[30] Nacoon S., Jogloy S., Riddech N., Mongkolthanaruk W., Kuyper T.W., Boonlue S. (2020). Interaction between Phosphate Solubilizing Bacteria and Arbuscular Mycorrhizal Fungi on Growth Promotion and Tuber Inulin Content of *Helianthus tuberosus* L.. Sci. Rep..

[31] Etesami H., Jeong B.R., Glick B.R. (2023). Potential use of *Bacillus* spp. as an effective biostimulant against abiotic stresses in crops—A review. Curr. Res. Biotechnol..

[32] Jankauskienė J., Mockevičiūtė R., Jurkonienė S., Gavelienė V., Buzytė K., Ustilaitė D., Todorova D. (2024). Microbial biostimulant counteracts negative effects of herbicides on oilseed rape growth. Sustain. Chem. Pharm..

[33] Baltazar M., Correia S., Guinan K.J., Sujeeth N., Bragança R., Gonçalves B. (2021). Recent Advances in the Molecular Effects of Biostimulants in Plants: An Overview. Biomolecules.

[34] Martínez-Martínez E., Slocum A.H., Ceballos M.L., Aponte P., Bisonó-León A.G. (2025). Beyond the Bloom: Invasive Seaweed *Sargassum* spp. as a Catalyst for Sustainable Agriculture and Blue Economy—A Multifaceted Approach to Biodegradable Films, Biostimulants, and Carbon Mitigation. Sustainability.

[35] Market.us (2025). Global Seaweed Extract Biostimulant Market Size.

[36] Sun W., Shahrajabian M.H., Kuang Y., Wang N. (2024). Amino Acids Biostimulants and Protein Hydrolysates in Agricultural Sciences. Plants.

[37] Pasković I., Andlovic M., Plešnik H., Vavpetič P., Žurga P., Popović L., Šala M., Franić M., Dlačić I., Ban S.G. (2026). Foliar Application of Protein Hydrolysates Promotes Growth and Affects Leaf Ionome in Olive. Horticulturae.

[38] Struszczyk-Świta K., Drożdżyński P., Marcinkowski P., Nadziejko A., Rodziewicz M., Januszewicz B., Gierszewska M., Marchut-Mikołajczyk O. (2025). Feather Waste Biodegradation and Biostimulant Potential of *Gordonia alkanivorans* S7: A Novel Keratinolytic Actinobacterium for Sustainable Waste Valorization. Int. J. Mol. Sci..

[39] Nuzhyna N., Raksha N., Halenova T., Vovk T., Savchuk O., Maievska T., Maievskyi K., Tonkha O., Ostapchenko L. (2024). Fish Hydrolysates as Potential Biostimulants for Growing Legumes and Cereals to Reduce Temperature Stress. Open Agric. J..

[40] Canellas L.P., da Silva R.M., Busato J.G., Olivares F.L. (2024). Humic substances and plant abiotic stress adaptation. Chem. Biol. Technol. Agric..

[41] Calvo P., Nelson L., Kloepper J.W. (2014). Agricultural uses of plant biostimulants. Plant Soil.

[42] Nardi S., Panuccio M., Abenavoli M., Muscolo A. (1994). Auxin-like effect of humic substances extracted from faeces of *Allolobophora caliginosa* and *A. rosea*. Soil Biol. Biochem..

[43] Qin K., Dong X., Joshi V., Lee C., Harvey J.T., Leskovar D.I. (2025). Biostimulant action of humic substances on tomato physiology and metabolism under water and nitrogen stresses. Plant Stress.

[44] Riseh R.S., Vatankhah M., Hassanisaadi M., Khandani Y., Skorik Y.A. (2026). Chitosan-based biostimulants for improving soil health, water and nutrient availability. Sci. Total Environ..

[45] Assali M., Sawalha S., Hamad R., Badran I., Eid A. (2025). Chitosan-functionalized amino acids as biostimulants for advancing sustainable agriculture. Int. J. Biol. Macromol..

[46] Kumaraswamy R.V., Kumari S., Choudhary R.C., Sharma S.S., Pal A., Raliya R., Biswas P., Saharan V. (2019). Salicylic acid functionalized chitosan nanoparticle: A sustainable biostimulant for plant. Int. J. Biol. Macromol..

[47] Ayub M.A., Abbas M., Rehman M.Z.U. (2023). Role of inorganic bio stimulant elements in plant growth. Sustainable Plant Nutrition.

[48] Kowalska I., Kowalczyk M., Mołdoch J., Pawelec S., Radzikowski P., Feledyn-Szewczyk B. (2025). The Effects of a Cultivar and Silicon Treatments on Grain Parameters and Bioactive Compound Content in Organic Spring Wheat. Foods.

[49] Di Mola I., Ottaiano L., Cozzolino E., Marra R., Vitale S., Pironti A., Fiorentino N., Mori M. (2023). Yield and Quality of Processing Tomato as Improved by Biostimulants Based on *Trichoderma* sp. and *Ascophyllum nodosum* and Biodegradable Mulching Films. Agronomy.

[50] Mousa E.-S.M., Elbagory M., Mahdy M.E., Abo-Koura H.A., Omara A.E.-D. (2024). Microencapsulation of *Bacillus megaterium* in Humic Acid-Supplied Alginate Beads Enhances Tomato Growth and Suppresses the Root-Knot Nematode *Meloidogyne javanica* Under Greenhouse Conditions. Horticulturae.

[51] Benito P., Celdrán M., Bellón J., Arbona V., González-Guzmán M., Porcel R., Yenush L., Mulet J.M. (2024). The combination of a microbial and a non-microbial biostimulant increases yield in lettuce (*Lactuca sativa*) under salt stress conditions by up-regulating cytokinin biosynthesis. J. Integr. Plant Biol..

[52] Rajalakshmi G., Balan S., K R., Sankaran G.B. (2025). Marine Derived Chitin as a Promising Bio-Stimulant for Sustainable Agriculture. Trout J. Atatürk Univ..

[53] Sible C.N., Seebauer J.R., Below F.E. (2025). Biostimulant or biological? The complexity of defining, categorizing, and regulating microbial inoculants. Agric. Environ. Lett..

[54] Izquierdo J., Arriagada O., García-Pintos G., Ortiz R., García-Pintos M. (2024). On-farm foliar application of a humic biostimulant increases the yield of rice. Agron. J..

[55] Nair A., Maity S., Pai V. (2025). Sustainable extraction: A comprehensive review of advancements beyond conventional methods. Microchem. J..

[56] Michalak I., Górka B., Wieczorek P.P., Rój E., Lipok J., Łęska B., Messyasz B., Wilk R., Schroeder G., Dobrzyńska-Inger A. (2016). Supercritical fluid extraction of algae enhances levels of biologically active compounds promoting plant growth. Eur. J. Phycol..

[57] Michalak I., Chojnacka K., Dmytryk A., Wilk R., Gramza M., Rój E. (2016). Evaluation of Supercritical Extracts of Algae as Biostimulants of Plant Growth in Field Trials. Front. Plant Sci..

[58] Michalak I., Tuhy Ł., Chojnacka K. (2015). Seaweed extract by microwave assisted extraction as plant growth biostimulant. Open Chem..

[59] Han S., Park J.-S., Umanzor S., Yarish C., Kim J.K. (2022). Effects of extraction methods for a new source of biostimulant from *Sargassum horneri* on the growth of economically important red algae, *Neopyropia yezoensis*. Sci. Rep..

[60] Szparaga A., Kocira S., Kapusta I., Zaguła G. (2021). Prototyping extracts from Artemisia absinthium L. for their biostimulating properties yield-enhancing, and farmer income-increasing properties. Ind. Crops Prod..

[61] Bhattacharya A., Mishra P., Mishra I., Arora P., Arora N.K. (2024). Microbe-based biostimulants: Latest developments and future perspectives. Microbial Biotechnology for Sustainable Agriculture.

[62] Shrivastava V., Edayilam N., Just B.S., Castaño-Sanchez O., Díaz-Guerra L., Meers E. (2024). Evaluation of agronomic efficiency and stress resistance on Swiss chard via use of biostimulants. Sci. Hortic..

[63] Inca-Torres R., Aguilera-Velázquez J.R., Urbina-Salazar A.d.R., Carbonero-Aguilar P., Navarro I.M., Palomas J.B. (2023). Enzymatic Preparation of Mushroom By-product Protein Hydrolysates (Mb-PPHs). Waste Biomass-Valorization.

[64] Goñi O., Quille P., O’Connell S. (2016). Production of chitosan oligosaccharides for inclusion in a plant biostimulant. Pure Appl. Chem..

[65] Mattedi A., Sabbi E., Farda B., Djebaili R., Mitra D., Ercole C., Cacchio P., Del Gallo M., Pellegrini M. (2023). Solid-State Fermentation: Applications and Future Perspectives for Biostimulant and Biopesticides Production. Microorganisms.

[66] Caruso G., De Pascale S., Cozzolino E., Giordano M., El-Nakhel C., Cuciniello A., Cenvinzo V., Colla G., Rouphael Y. (2019). Protein Hydrolysate or Plant Extract-based Biostimulants Enhanced Yield and Quality Performances of Greenhouse Perennial Wall Rocket Grown in Different Seasons. Plants.

[67] Porras R.C.S., Ghoreishi G., Sánchez A., Barrena R., Font X., Ballardo C., Artola A. (2025). Solid-state fermentation of green waste for the production of biostimulants to enhance lettuce (*Lactuca sativa* L.) cultivation under water stress: Closing the organic waste cycle. Chemosphere.

[68] Singh V., Bhat R.A., Dar G.H. (2024). Nanobiostimulants: Emerging Strategies for Agricultural Sustainability.

[69] Naheed N., Shahzad A., Jabeen A., Umer M., Ilyas N. (2025). Microbial Nano-biostimulants for Improved Crop Productivity. Nanobiostimulants in Innovative Agriculture.

[70] Zhang Q., Ahmed T., Noman M., Qi Y., Ijaz M., Li Z., Yang H., Sun L., Qi X., Li B. (2025). Immunomodulatory nano-biostimulants remodel transcriptome and metabolome for enhancing bayberry resilience against twig blight disease. Chem. Eng. J..

[71] Khepar V., Sidhu A., Mankoo R.K., Manchanda P., Sharma A.B. (2024). Nanobiostimulant action of trigolic formulated zinc sulfide nanoparticles (ZnS-T NPs) on rice seeds by triggering antioxidant defense network and plant growth specific transcription factors. Plant Physiol. Biochem..

[72] Cozzolino E., Di Mola I., Ottaiano L., El-Nakhel C., Rouphael Y., Mori M. (2021). Foliar application of plant-based biostimulants improve yield and upgrade qualitative characteristics of processing tomato. Ital. J. Agron..

[73] Vignesh M., Manjusha M.R., Bharathi S., Dharani J., Gowshika R., Gobika C., Anchana K., Devi E.S., Aneesha J., Udhayakumar K. (2025). Foliar Spray of Biostimulants as a Sustainable Strategy to Enhance Growth and Floral Productivity of Marigold (*Tagetes erecta* L.). Int. J. Bio-Resour. Stress Manag..

[74] Soppelsa S., Kelderer M., Casera C., Bassi M., Robatscher P., Matteazzi A., Andreotti C. (2019). Foliar Applications of Biostimulants Promote Growth, Yield and Fruit Quality of Strawberry Plants Grown under Nutrient Limitation. Agronomy.

[75] Abdón-Aguilar G., Rueda-Altunar A.L., Robledo-Olivo A., González-Morales S., Benavides-Mendoza A., Charles-Rodríguez A.V., Juárez-Maldonado A., la Fuente M.C.-D. (2026). Foliar Application of a Biostimulant Based on Fermented Pomegranate Waste Increases Tomato Yield in Greenhouse. Scientifica.

[76] Ali O., Ramsubhag A., Jayaraman J. (2021). Biostimulant Properties of Seaweed Extracts in Plants: Implications towards Sustainable Crop Production. Plants.

[77] Papnai N., Chaurasiya D.K., Sahni S. (2022). Biostimulants: Concept, Types and Way to Enhance Soil Health. Int. J. Plant Soil Sci..

[78] Li J., Van Gerrewey T., Geelen D. (2022). A Meta-Analysis of Biostimulant Yield Effectiveness in Field Trials. Front. Plant Sci..

[79] Youssef S.M., El-Serafy R.S., Ghanem K.Z., Elhakem A., Aal A.A.A. (2022). Foliar Spray or Soil Drench: Microalgae Application Impacts on Soil Microbiology, Morpho-Physiological and Biochemical Responses, Oil and Fatty Acid Profiles of Chia Plants under Alkaline Stress. Biology.

[80] Wang Z., Yang R., Liang Y., Zhang S., Zhang Z., Sun C., Li J., Qi Z., Yang Q. (2022). Comparing Efficacy of Different Biostimulants for Hydroponically Grown Lettuce (*Lactuca sativa* L.). Agronomy.

[81] Habibi N., Terada N., Pachakkil B., Sanada A., Kamata A., Koshio K. (2025). Effect of Priming Treatment on Improving Germination and Seedling Performance of Aged and Iron-Coated Rice Seeds Aiming for Direct Sowing. Plants.

[82] Haider N., Noor A., Zeb M.M., Abro M., Hussain S., Baloch G., Khoso A.R., Wahocho S.A. (2024). Evaluating the effect of priming with moringa leaf extract on seed germination and seedling growth of bitter gourd (*Momordica charantia* L). J. Hortic. Agric. Sci..

[83] Kumari R., Sudhanshu K., Priyanka K., Vikash K., Tushar R., Anand K., Vandita K., Subrat K.B., Bineeta M.B., Ravi R.K. (2025). Seed priming with chitosan and its nanoparticles improve physiological, biochemical and crop performances in lentil (*Lens culinaris* L.). Plant Sci. Today.

[84] Han M., Kasim S., Yang Z., Deng X., Saidi N.B., Uddin K., Shuib E.M. (2024). Plant Extracts as Biostimulant Agents: A Promising Strategy for Managing Environmental Stress in Sustainable Agriculture. Phyton.

[85] Lutts S., Benincasa P., Wojtyla L., Kubala S.S., Pace R., Lechowska K., Quinet M., Garnczarska M. (2016). Seed Priming: New Comprehensive Approaches for an Old Empirical Technique. New Challenges in Seed Biology-Basic and Translational Research Driving Seed Technology.

[86] Omoarelojie L.O., Kulkarni M.G., Finnie J.F., Van Staden J. (2021). Modes of action of biostimulants in plants. Biostimulants for Crops from Seed Germination to Plant Development.

[87] Trevisan S., Pizzeghello D., Ruperti B., Francioso O., Sassi A., Palme K., Quaggiotti S., Nardi S. (2010). Humic substances induce lateral root formation and expression of the early auxin-responsive IAA19 gene and DR5 synthetic element in Arabidopsis. Plant Biol..

[88] Lopez-Moya F., Escudero N., Zavala-Gonzalez E.A., Esteve-Bruna D., Blázquez M.A., Alabadí D., Lopez-Llorca L.V. (2017). Induction of auxin biosynthesis and WOX5 repression mediate changes in root development in Arabidopsis exposed to chitosan. Sci. Rep..

[89] Pizzeghello D., Francioso O., Ertani A., Muscolo A., Nardi S. (2013). Isopentenyladenosine and cytokinin-like activity of different humic substances. J. Geochem. Explor..

[90] Stanga J.P., Smith S.M., Briggs W.R., Nelson D.C. (2013). Suppressor of more axillary growth2 1 controls seed germination and seedling development in Arabidopsis. Plant Physiol..

[91] Wally O.S.D., Critchley A.T., Hiltz D., Craigie J.S., Han X., Zaharia L.I., Abrams S.R., Prithiviraj B. (2013). Regulation of Phytohormone Biosynthesis and Accumulation in Arabidopsis Following Treatment with Commercial Extract from the Marine Macroalga Ascophyllum nodosum. J. Plant Growth Regul..

[92] Ertani A., Cavani L., Pizzeghello D., Brandellero E., Altissimo A., Ciavatta C., Nardi S. (2009). Biostimulant activity of two protein hydrolyzates in the growth and nitrogen metabolism of maize seedlings. J. Plant Nutr. Soil Sci..

[93] Schiavon M., Pizzeghello D., Muscolo A., Vaccaro S., Francioso O., Nardi S. (2010). High Molecular Size Humic Substances Enhance Phenylpropanoid Metabolism in Maize (*Zea mays* L.). J. Chem. Ecol..

[94] De Saeger J., Van Praet S., Vereecke D., Park J., Jacques S., Han T., Depuydt S. (2020). Toward the molecular understanding of the action mechanism of Ascophyllum nodosum extracts on plants. J. Appl. Phycol..

[95] Quaggiotti S., Ruperti B., Pizzeghello D., Francioso O., Tugnoli V., Nardi S. (2004). Effect of low molecular size humic substances on nitrate uptake and expression of genes involved in nitrate transport in maize (*Zea mays* L.). J. Exp. Bot..

[96] Goñi O., Fort A., Quille P., McKeown P.C., Spillane C., O’cOnnell S. (2016). Comparative Transcriptome Analysis of Two *Ascophyllum nodosum* Extract Biostimulants: Same Seaweed but Different. J. Agric. Food Chem..

[97] Aamir M., Kashyap S.P., Zehra A., Dubey M.K., Singh V.K., Ansari W.A., Upadhyay R.S., Singh S. (2019). Trichoderma erinaceum Bio-Priming Modulates the WRKYs Defense Programming in Tomato Against the *Fusarium oxysporum* f. sp. lycopersici (Fol) Challenged Condition. Front. Plant Sci..

[98] Shukla P.S., Borza T., Critchley A.T., Hiltz D., Norrie J., Prithiviraj B. (2018). Ascophyllum nodosum extract mitigates salinity stress in Arabidopsis thaliana by modulating the expression of miRNA involved in stress tolerance and nutrient acquisition. PLoS ONE.

[99] Di Sario L., Boeri P., Matus J.T., Pizzio G.A. (2025). Plant Biostimulants to Enhance Abiotic Stress Resilience in Crops. Int. J. Mol. Sci..

[100] Moustakas M., Moustaka J. (2026). Special Issue: “Molecular Mechanisms of Plant Biostimulants”. Int. J. Mol. Sci..

[101] Nephali L., Piater L.A., Dubery I.A., Patterson V., Huyser J., Burgess K., Tugizimana F. (2020). Biostimulants for Plant Growth and Mitigation of Abiotic Stresses: A Metabolomics Perspective. Metabolites.

[102] Mazur Ł., Kamecki B., Ignaczak J., Molin S., Brylewski T. (2025). Performance of Ni-and Fe-Doped Cu–Mn Spinel Coatings Enhanced with Nanoparticles for SOEC Interconnects. Electrochemical Society Meeting Abstracts sofc2025.

[103] Franzoni G., Cocetta G., Prinsi B., Ferrante A., Espen L. (2022). Biostimulants on Crops: Their Impact under Abiotic Stress Conditions. Horticulturae.

[104] Belal H.E.E., Elkelish A., Zaid M.M., Alhudhaibi A., El-Roby M.S.A., Elmohsen Y.H.A., Abeed A.H.A., Ukozehasi C., Rady M.M., Sayed A.A.S. (2025). Novel biostimulants-mediate tolerance to drought stress in Phaseolus vulgaris plants by optimizing osmoprotectants and antioxidant defense systems. Bot. Stud..

[105] Kumar V.Y., Kavitha S., Boominathan P., Manonmani V., Hemavathy A.T., Malarkodi K., Sudha A., Pradipa C. (2025). Boosting crop productivity: The essential role of biostimulants under abiotic stress conditions. Plant Sci. Today.

[106] Punjamgod D., Kurinjery A., Annamalai M., Rathinam R., Kulanthaiyesu A. (2026). Structural diversity, biosynthesis, and extraction of brown algae fucoidan and its bio-stimulant applications in crop improvement. Crit. Rev. Biotechnol..

[107] Szparaga A., Kocira S., Kapusta I., Zaguła G. (2023). Solid–liquid extraction of bioactive compounds as a green alternative for developing novel biostimulant from *Linum usitatissimum* L.. Chem. Biol. Technol. Agric..

[108] Pompermaier M., Ambrosini S., Zamboni A., Bolzonella D., Pesante G. (2025). A biorefinery approach for biostimulants and PHAs production from agri-food waste: Improved treatment strategies. Environ. Technol. Innov..

[109] Smidt-Jensen A., Røgild T.B., Cohen T., Meshoulam S., Iuclea L., Sigurjónsson H.Æ., Tzachor A., Geirsdóttir M., Moomaw W.R. (2026). Life Cycle Assessment of Phycocyanin Food Colorant Production from Spirulina (*Arthrospira platensis*) with Biostimulant Waste-Stream Utilization for Soil Carbon Sequestration to Achieve Net Carbon Removal. Foods.

[110] Rafya M., Benkhalti F., Zehhar N. (2026). Efficacy of rosemary water residue as a biostimulant through foliar spray for tomato cultivation: Molecular docking and Life cycle assessment. Biocatal. Agric. Biotechnol..

[111] (2019). EUR 29841 EN; Technical Proposals for Selected New Fertilising Materials Under the Fertilising Products Regulation (Regulation (EU) 2019/1009). https://publications.jrc.ec.europa.eu/repository/handle/JRC117856.

[112] Santos F., Melkani S., Oliveira-Paiva C., Bini D., Pavuluri K., Gatiboni L., Mahmud A., Torres M., McLamore E., Bhadha J.H. (2024). Biofertilizer use in the United States: Definition, regulation, and prospects. Appl. Microbiol. Biotechnol..

[113] (2021). Gazette of India, Part II, Section 3, Sub-Section (ii); The Fertiliser (Inorganic, Organic or Mixed) (Control) Sixth Amendment Order. https://www.legitquest.com/act/fertiliser-inorganic-organic-or-mixed-control-amendment-order-2021/CAFF.

[114] (2017). Decree No. 32; Measures for the Registration of Fertilizers. http://en.zbhegui.com/item/zgfldjdl.html.

[115] (2012). Composts, Soil Conditioners and Mulches.

[116] (2020). Instrução Normativa Nº 61, de 8 de julho de 2020; Rules on the Definitions, Requirements, Specifications, Guarantees, Tolerances, Registration, Packaging and Labeling of Organic Fertilizers and Biofertilizers. https://www.gov.br/agricultura/pt-br/assuntos/insumos-agropecuarios/insumos-agricolas/fertilizantes/legislacao/in-61-de-8-7-2020-organicos-e-biofertilizantes-dou-15-7-20.pdf#:~:text=Estabelece%20as%20regras%20sobre%20defini%C3%A7%C3%B5es%2C,dos%20fertilizantes%20org%C3%A2nicos%20e%20dos.

[117] Sharifi L., Ghiyasi M., Talebian B., Danesh Y.R., Najafi S., Tunçtürk M., Tunçtürk R., Farda B., Pace L.G. (2026). Machine learning-based evaluation of seed priming and biostimulant applications in rainfed wheat. PeerJ.

[118] Lucini L., Rouphael Y., Cardarelli M., Bonini P., Baffi C., Colla G. (2018). A Vegetal Biopolymer-Based Biostimulant Promoted Root Growth in Melon While Triggering Brassinosteroids and Stress-Related Compounds. Front. Plant Sci..

[119] Lephatsi M., Nephali L., Meyer V., Piater L.A., Buthelezi N., Dubery I.A., Opperman H., Brand M., Huyser J., Tugizimana F. (2022). Molecular mechanisms associated with microbial biostimulant-mediated growth enhancement, priming and drought stress tolerance in maize plants. Sci. Rep..

[120] Pai D.G., Balachandra M., Kamath R. (2025). Explainable AI in agriculture: Review of applications, methodologies, and future directions. Eng. Res. Express.

[121] Dhotre A.D., Thorat S.A., Yelure B.S., Jawade P.B. (2026). An Explainable AI-Driven Framework for Precision Agriculture: A Comprehensive Survey. Agron. Res..

[122] Rouphael Y., Colla G. (2020). Biostimulants in agriculture. Front. Plant Sci..

[123] Giordano M., El-Nakhel C., Carillo P., Colla G., Graziani G., Di Mola I., Mori M., Kyriacou M.C., Rouphael Y., Soteriou G.A. (2022). Plant-Derived Biostimulants Differentially Modulate Primary and Secondary Metabolites and Improve the Yield Potential of Red and Green Lettuce Cultivars. Agronomy.

[124] Mulya K.S., Tan J.P., Yeat S.P., Yeat C.N.C., Woon K.S. (2024). Biofertilizers for Sustainable Agriculture: A Life Cycle Assessment of Upstream Manufacturing to Carbon Reduction. Chem. Eng. Trans..

[125] Jacomassi L.M., Viveiros J.d.O., Oliveira M.P., Momesso L., de Siqueira G.F., Crusciol C.A.C. (2022). A Seaweed Extract-Based Biostimulant Mitigates Drought Stress in Sugarcane. Front. Plant Sci..

[126] Gold Standard (2023). Soil Organic Carbon Activity Module: Biostimulants for Soil Revitalization.

[127] European Parliament and Council of the European Union (2024). Regulation (EU) 2024/3012 of the European Parliament and of the Council of 27 November 2024 Establishing a Union Certification Framework for Permanent Carbon Removals, Carbon Farming and Carbon Storage in Products. Official Journal of the European Union, OJ L 2024/3012. https://eur-lex.europa.eu/eli/reg/2024/3012/oj/eng.

[128] Bartucca M.L., Cerri M., Del Buono D., Forni C. (2022). Use of Biostimulants as a New Approach for the Improvement of Phytoremediation Performance—A Review. Plants.

[129] du Jardin P., Brown P.H., DeJong T.M., Cassán F., Ferrante A., Fotopoulos V., Manganaris G.A., Carillo P. (2025). Unlocking the black box of plant biostimulants. Sci. Hortic..

[130] Wang Y., Xiong H., Zhou L., Sun Y., Yang J., Shi X., Zhang Y., Zhang F., Rennenberg H. (2026). Biostimulant Applications Improve Crop Root Morphology in Agricultural Systems: A Global Meta-Analysis. Agronomy.

